# Resveratrol-Linoleate protects from exacerbated endothelial permeability via a drastic inhibition of the MMP-9 activity

**DOI:** 10.1042/BSR20171712

**Published:** 2018-07-31

**Authors:** Aly Shamseddin, Céline Crauste, Erwan Durand, Pierre Villeneuve, Grégor Dubois, Teresa Pavlickova, Thiery Durand, Joseph Vercauteren, Francisco Veas

**Affiliations:** 1 Molecular Comparative Immuno-Physiopathology Lab (LIPMC), French Institute of Research for Development (IRD), Joint Research Unit-Ministry of Defense (UMR-MD), Faculty of Pharmacy, Montpellier University, 34093 Montpellier, France; 2 Institute for Biomolecules Max Mousseron (IBMM), UMR 5247 CNRS, UM, ENSCM, Faculty of Pharmacy, Laboratory of Pharmacognosy, Montpellier University, 34093 Montpellier, France; 3 International French Center for Agronomy Research (CIRAD), UMR-IATE, F-34060, Montpellier, France; 4 Faculty of Biotechnology, Misr University for Science and Technology (MUST), PO Box 77, Giza, Egypt

**Keywords:** endothelial dysfunction, Inflammation, MMP-9, Natural deep eutectic solvent (NADES), resveratrol-linoleate, vascular leakage

## Abstract

Gelatinolytic matrix metalloproteinases (MMP-2, -9) play a critical role not only in mammals physiology but also during inflammation and healing processes. The natural stilbenoid, resveratrol (RES), exhibits potent antioxidant effects, in a hormetic mode of action, and is known to inhibit MMP-9. However, RES administration exhibits major issues, including poor bioavailability and water solubility, hampering its potential therapeutic effect *in vivo*. In the present study, we synthesized and evaluated five novel RES–lipid conjugates to increase their cell membrane penetration and improve their bioavailability. The best *in vitro* MMP-9 inhibitory activity of RES–lipids conjugates was observed with RES-linoleic acid (LA) (5 µM), when dissolved in a natural deep eutectic solvent (NADES), composed of an equimolar content of 1,2-propanediol:choline chloride (ChCl):water. The inhibition of MMP-9 expression by RES-LA in activated THP-1 monocytes, was, at least due to the deactivation of ERK1/2 and JNK1/2 MAP kinase signaling pathways. Moreover, RES-LA exhibited a strong effect protecting the TNF-α-induced exacerbated permeability in an HUVEC *in vitro* monolayer (by 81%) via the integrity protection of intercellular junction proteins from the MMP-9 activity. This effect was confirmed by using several complementary approaches including, the real-time monitoring of trans-endothelial electric resistance (TEER), the Transwell HUVEC permeability level, the microscopic examination of the platelet endothelial cell adhesion molecule-1 (CD31/PECAM-1) integrity as well as the fluorescence in intercellular spaces. Consequently, following this strong *in vitro* proof-of-concept, there is a need to test this promising RES–lipid derivative compound to control the pathological endothelial permeability *in vivo*.

## Introduction

Matrix metalloproteinases (MMPs) are members of a family composed of at least six different subfamilies with more than 25 zinc-dependent endopeptidases, which function in the extracellular environment and degrade both matrix and non-matrix proteins [1]. In particular, the gelatinolytic MMPs, mainly MMP-9, and to a lesser extent MMP-2, play a key role in the pathogenesis process of several diseases, through a degradation of intercellular junction proteins, resulting in an exacerbated increase in the vascular permeability [2,3]. While several studies have established correlations between the overproduction of active MMP-9 and the pathological exacerbated vascular permeability, some reports have evidenced that inhibitors of MMP-9 could play a mitigating role in the progression of the MMP-9-associated pathogenesis. Despite several pre-clinical and clinical trials to test the safety and efficacy of newly designed MMP-9 inhibitors, none of them have been clinically approved, due to their poor bioavailability and/or toxicity [4,5]. Consequently, the production of efficient, safe, and well-formulated compounds to control the pathological MMP-9 activity is still one of the major clinical unmet needs. Therefore, in this preliminary *in vitro* study, we jointly explored two functional aspects of the question, i.e. the potential therapeutic effect of a new MMP-9 inhibitor and a new formulation, which remains another major issue to be solved.

Resveratrol (RES) is a natural polyphenolic phytoalexin mainly found in *Vitis vinifera* (Vitaceae) stalks and in the roots of *Fallopia japonica* var. *japonica* (Polygonaceae). Despite that RES is well known for its antioxidant, anti-inflammatory, and antiviral effects, and to a lesser extent for its inhibitory activity on active gelatinolytic MMP, notably MMP-9 [6–8]. However, its clinical use remains limited due to its poor bioavailability [9,10]. In our previous report on phloroglucinol (a true vinylogous of RES) [11], we prepared lipophenolic derivatives of RES using a lipid moiety such as polyunsaturated fatty acids (PUFA), prone to protect its phenolic functional group from rapid metabolization and excretion and to increase its ability to penetrate and cross lipid membranes (increasing lipophilicity). Herein, four new RES lipophenols were synthesized and evaluated for their capacity to inhibit MMP-9.

Natural deep eutectic solvents (NADES), recently discovered by Choi and colleagues [12], are considered as a third class of plant solvents, in addition to water and oil phases. They mainly consist of primary plant metabolites, including organic hydroxyacids, amino acids, and sugars. As soon as these metabolites are mixed together in a specific molar ratio, they remain in a liquid state at a much lower temperature than their individual components. The emerging use of this new solvent class has been particularly observed in the field of green chemistry, for the extraction of different plant bioactive compounds and much less applied to the extraction of organic solvents (which are more toxic and volatile) [13]. In our previous study [14], we found that a particular NADES, consisting of an equimolar 1,2-propanediol:choline chloride:water mix (PCW), was not only able to solubilize RES, but also to increase its capacity to inhibit MMP-9 activity, by at least a ten-fold factor as compared with DMSO. Thus, NADES-PCW enabled us to reach the RES hormetic mode of action. Therefore, we also used NADES in the present study for the evaluation of the MMP-9-inhibitory activity of RES-linoleic acid (LA).

## Materials and methods

### Cells and reagents

THP-1 monocytes were cultured in 10% heat-inactivated FBS RPMI-1640 medium supplemented with penicillin-G 100 units/ml and streptomycin 100 μg/ml. HUVEC were cultured in (1%) low serum EndoGro™ medium, culture kit purchased from Merck Millipore™ (Paris, France). Micro-BCA Protein Assay Kit was purchased from Fisher Scientific™ (Illkirch-Graffenstaden, France). TNF-α was purchased from PeproTech™ (Neuilly-sur-Seine, France). Non-conjugated guinea pig anti-MMP-9 EP1255Y mAb was purchased from antibodies-online (Aachen, Germany). The cy3-conjugated human anti-CD31/PECAM-1 (platelet endothelial cell adhesion molecule-1) mAb (clone 9G11), and streptavidin-fluorescein were purchased from bio-Techne™ (Abingdon, United Kingdom). SB-3CT (commercial MMP-2, -9 inhibitor) was purchased from Enzo Life Sciences™ (Villeurbanne, France). RES was purified from stalks of *Vitis vinifera*, Vitaceae, according to the process described by Delaunay and colleagues [26]. JNK inhibitor (sp600125), DMSO, MTT (Thiazolyl Blue), all chemicals and solvents used during the synthesis individual NADES components were purchased from Sigma–Aldrich™ (Marnes la Coquette, France). All other chemicals used in the present study are of molecular biology grade.

### Synthesis of RES-derived lipophenols

**Figure 1:**
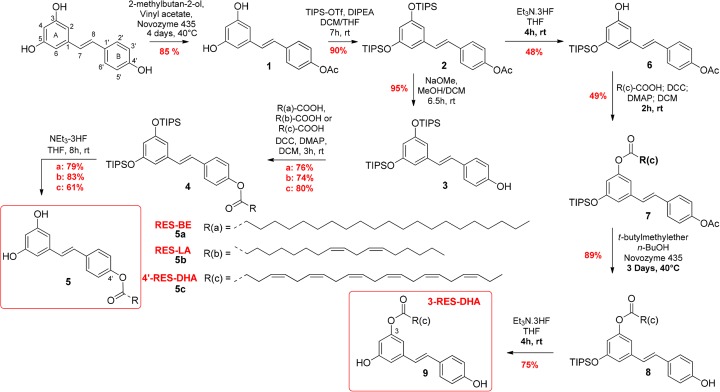
Synthesis of RES-lipophenol derivatives

To evaluate the activity of different lipid chains at the 4′ position, RES-behenic acid (BE) (compound #**5a**), RES-LA (compound #**5b**), and RES-docosahexaenoic acid (DHA) (compound #**5c**), were synthesized using enzymatic and chemical synthesis starting from RES (Figure 1). In the first step, a lipase, from *Candida antarctica* (CALB, Novozyme 435, selective of the 4′ position) was used to regio-selectively introduce the acetyl group at the RES C4′-OH position. The reaction was performed with good yield (85%) without any acetylation at the position 3/5. Hydroxyl groups at positions 3 and 5 of the compound #**1** were thus protected by triisopropyl silyl (TIPS) protecting groups using triflate reagent (TIPS-OTf) and diisopropylethylamine (DIPEA) as a base to obtain the protected derivative (compound #**2)**. The acetyl group of the compound #**2** was deprotected with a solution of sodium methanolate (MeONa) in anhydrous methanol and resulted in RES-diTIPS (compound #**3**), with a yield of 95%. The coupling reactions between compound #**3** and the different fatty acids (FA), BE, LA, and DHA, were initiated using dicyclohexylcardodiimide and dimethylamino pyridine (DCC/DMAP) as coupling reagents to access compounds #**4a–c**. Final deprotection of TIPS protecting groups by Et_3_N-3HF in dry tetrahydrofuran (THF) yielded final lipophenols compounds #**5a–c.**

To study the importance of the position of the lipidic part on the RES structure, we synthesized a lipidic RES having FA at the position 3. Starting from the fully protected RES (compound #**2**), one TIPS group at the position 5 was removed using mild Et_3_N-3HF carefully monitored by TLC (48%, Figure 1). Afterward, the mono-deprotected derivative **6** was linked to the FA (DHA) using DCC/DMAP. In order to preserve the ester linkage of the compound **7**, the acetate group was deprotected using enzymatic lipase CALB in the presence of butanol (89%) instead of MeONa solution. The final TIPS deprotection using Et_3_N-3HF provided the desired 3-RES-DHA (compound # **9**). A quality control assessment was established by a complete ^1^H and ^13^C NMR spectral analysis for each synthesized compound (Supplementary Information).

### Preparation of NADES

The NADES composed of the equimolar ratio of 1,2-propanediol, choline chloride, and water was prepared according to the protocol modified from Abbott and colleagues [27]. The mixture was incubated at 60°C and stirred with an orbital shaker at 300 rpm until forming a clear solution. The formed NADES was used as a solvent for RES and RES-LA at a final concentration of 10 mg/ml.

### Assessment of MMP-9 activity in THP-1 monocytes

TNF-α-activated THP-1 monocytes (3 × 10^5^ cells/ml) were seeded in 1% FBS RPMI-1640 to assess MMP-9 activity. First, RES and its derived lipophenolic compounds were dissolved in DMSO and incubated at a final concentration of 30 µM with 10 ng/ml TNF-α-activated THP-1 in a humid CO_2_ incubator chamber for 24 h at 37°C. Subsequently, the MMP-9 inhibitory activity in increasing concentrations (1, 5, 8, 10, 20, 30, and 40 µM) of RES-LA dissolved in NADES was tested on activated THP-1 monocytes in the same conditions mentioned above. After incubation, the cell suspension was centrifuged at 1000 rpm for 5 min; the supernatant was recovered and stored at −80°C for zymogram analyses. For the assessment of MMP-9 activity using JNK inhibitor (sp600125), cells were treated in the same conditions as mentioned above.

### Zymography

The inhibitory MMP-9 activity was assessed using gelatin zymography. Briefly, collected supernatants were loaded on SDS/PAGE (10% gel) supplemented with 1% gelatin without reducing agents. After separation, gels were incubated once with 2.5% Triton X-100, washed thrice for 5 min each with gelatinase buffer (NaCl 200 mM, Tris base 50 mM, CaCl_2_ 5 mM, and ZnCl_2_ 0.25 mM; pH 7.5) and incubated for 24 h in the same buffer at 37°C. Gels were further stained for 1 h with Coomassie Blue staining solution (0.025% Coomassie Blue, 40% methanol, and 10% acetic acid) followed by destaining with 20% methanol and 10% glacial acetic acid solution. Gel bands were photographed and analyzed using the GelAnalyzer 2010a™ software.

### MTT assay

The cytotoxicity assay was carried out as described by Mosmann [28]. Briefly, 100 µl of primary HUVEC and THP-1 monocytes (10^5^ cells/ml) were seeded in a 96-well plate and incubated in a CO_2_ chamber at 37°C for 24 h. Cells were treated for 72 h with 10, 20, 40, or 80 μM final concentration of RES-LA/NADES formulation. Cell medium was replaced with MTT medium and plates were incubated for three additional hours. The formed formazan blue crystals were further dissolved by adding 100 µl of 10% SDS in 0.01 N HCl to each well. The optical density was measured at wavelength 570 nm (against the reference at 690 nm) using a TECAN™ plate reader (Paris, France).

### Endothelial monolayer permeability assay

The permeability of the HUVEC monolayer was examined using the *in vitro* vascular permeability assay kit from Millipore™ (Paris, France). Briefly, HUVEC (3 × 10^5^ cells/insert) were seeded on collagen-coated semipermeable inserts for 48 h or until formation of a confluent monolayer. Subsequently, medium was replaced and TNF-α (100 ng/ml) was added to each insert with or without 10 µM of either RES, RES-LA, or SB-3CT, followed by 24-h incubation. The medium was replaced and FITC in cell culture medium (1:40) was added to each insert, and plates were incubated for 20 min in the dark. Absorbances were read on a TECAN™ spectrophotometer at 485–535 nm.

### Immunofluorescence

Primary HUVEC (3 × 10^5^ cells/well) were incubated on to biotinylated gelatin-coated slide chambers for 48 h up to their confluence permitting the establishment of intercellular junctions. Then, cells were treated with TNF-α 100 ng/ml with or without 10 µM RES, RES-LA, or SB-3CT. After 24 h, cells were fixed with 3.7% formaldehyde and washed thrice with PBS. Each chamber was incubated for 1 h with either FITC-conjugated streptavidin to stain the biotinylated-gelatin and therefore assess the intercellular spaces, or Cy3-conjugated anti-PECAM-1 mAb (8 µg/ml), in order to assess the integrity of intercellular junctions. After washing, slides were protected with mounting medium to be examined microscopically.

### Trans-endothelial electric resistance

The real-time permeability measurements of HUVEC monolayers were assayed using ECIS^™^ 8W10E+ arrays (Applied Biophysics, Troy, NY). HUVEC (2 × 10^5^ cells/well) were seeded in each gelatin-coated chamber and incubated for 48 h. After the formation of confluent endothelial monolayer, 10 µM of RES, RES-LA, or SB-3CT were incubated with 100 ng/ml TNF-α. Positive (TNF-α) and negative controls (0.1% NADES or DMSO) were also monitored, and cells were incubated for additional 48 h. The trans-endothelial electric resistance (TEER) measurements were done in real-time, and the resistance (Ω) was monitored at 8-s frequency measurement. Steady basal resistance fluctuations indicate healthy HUVEC monolayers [29].

### Real-time reverse transcription-PCR

Total RNA was isolated using TRIzol (Invitrogen). Briefly, RNA was extracted from TNF-α (10 ng/ml) activated THP-1 monocytes (7.5 × 10^5^ cells/well) with or without 10 µM of RES or RES-LA. Reverse transcription was done with 200 ng RNA per sample using Thermo Scientific RT-kit (random hexamer primer) and RT-MLV (Invitrogen). The cDNA was further diluted ten times to carry out a real-time reverse transcription (RT)-PCR. The primers used in the present study are: MMP-9 forward 5′-TTATCGCCGACAAGTGGCCCG-3′ and MMP-9 reverse 5′-AACTCGTCATCGTCGAAATGGGC-3′; HMBS forward 5′-TCACCATCGCAGCCATCT-3′; HMBS reverse 5′-GTTCCCACCACGCTCTTCT-3′. Relative quantities of amplicons from both MMP-9 and the housekeeping (HMBS) genes were calculated using the comparative threshold cycle number (*C*_T_) method from LightCycler 480™ device (Roche).

### Phospho-kinase cell signaling

The phosphorylation status of MAPKs (ERK, JNK, p38), Akt, MKK, and mTOR were examined using the Human Phopsho-MAPK Array (R&D Systems™, Minneapolis, U.S.A.). The experiment was started with 1-h pre-incubation of THP-1 monocytes (5 × 10^5^ cells/well) with 10 µM of either RES or RES-LA dissolved in NADES, followed by a 5-min incubation with 10 ng/ml TNF-α. Total protein content was quantitated, and 40 µg of cell lysate was used in the Western blot test. Detection of phosphorylated proteins was done using the kit-specific antibodies, that were revealed by chemiluminescent signal capture assay using a secondary horseradish peroxidase (HRP)-conjugated antibody (Jackson ImmunoResearch™, PA). Electrophoretically migrated proteins were analyzed using the GelAnalyzer software 2010a™.

### Statistical analysis

Data were expressed as mean ± S.D. and analyzed one-way and two-way ANOVA. The difference between groups was calculated using Tukey multiple comparison tests. A *P*-value below 0.05 was considered statistically significant. All experiments in the present study were repeated at least three times.

## Results

### Assessment of the MMP-9 inhibitory activity of RES derivatives

Four RES-lipophenolic derivatives were prepared and dissolved in DMSO: RES-BE, RES-LA, 3-RES-DHA, and 4′-RES-DHA (Figure 1). The hydrophilic/lipophilic balance was fine-tuned by esterification of RES by various FA, including the saturated derivative (BE, C22:0, RES-BE; compound #**5a**), omega-6 PUFA (LA, C18:2 n-6, RES-LA; compound #**5b**), and omega-3 PUFA (DHA, C22:6 n-3, RES-DHA; compound #**5c**) (Figure 1).

**Figure 2:**
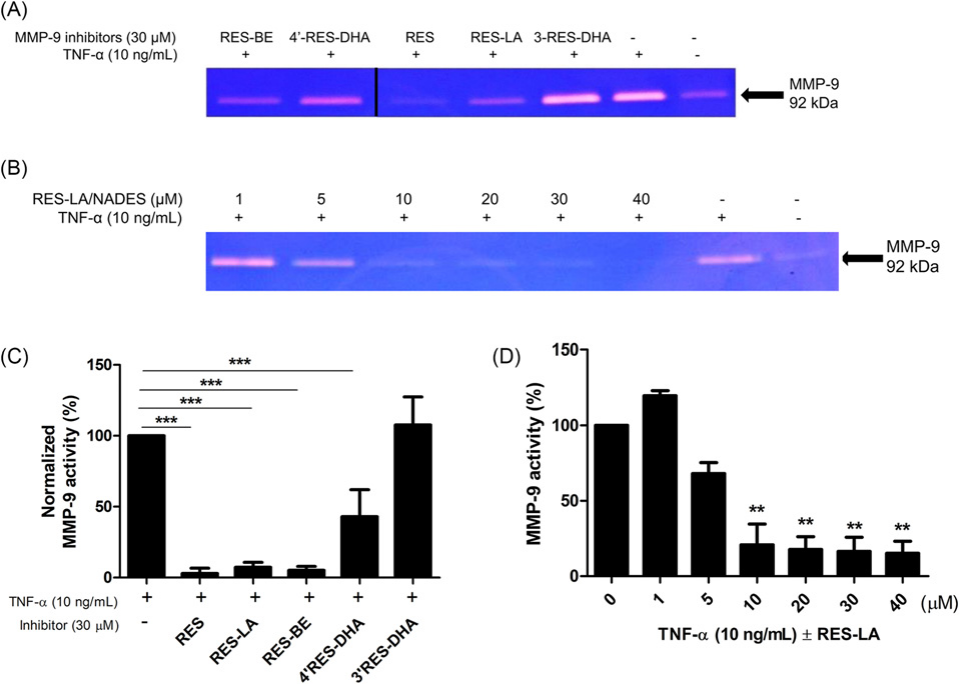
Assessment of MMP-9 inhibitory activity of RES derivatives THP-1 monocytes were seeded (3 × 10^5^ cells/well) in 24-well plates in the presence of TNF-α 10 ng/ml and incubated with RES derivatives at 30 µM (**A**), or with increasing concentrations (1, 5 10, 20, 30, 40 µM) of RES-LA (**B**). After an incubation lapse-time of 24 h at 37°C, the supernatant was collected and tested by zymography. Gel bands showed the MMP-9 gelatinolytic activity. Gel bands were analyzed, and data were plotted against TNF-α-treated groups (**C**,**D**). Data represent a mean ± S.D. **P≤0.01, ***P≤0.001.

Their MMP-9 inhibitory activity was then compared with RES and assessed *in vitro* on TNF-α-activated THP-1 monocytes. Our results showed that RES, RES-LA, RES-BE and to a lesser extent 4′-RES-DHA exhibited an MMP-9-inhibitory activity in TNF-α-activated monocytes (Figure 2A). Despite that RES-BE has demonstrated MMP-9 inhibitory active, the addition of a saturated BE in the 4′ position of RES highly decreases its solubility. Alternatively, 30 µM RES-DHA was able to reduce MMP-9 activity, its regio-isomer, having the lipid chain linked with the hydroxyl group in position 3 of RES (3-RES-DHA = compound #**9**), was not active at the same concentration.

After the selection of RES-LA (based upon its better solubility and activity), the compound was dissolved in NADES-PCW, and a dose–response MMP-9 inhibitory activity experiment was carried out on TNF-α-activated THP-1 monocytes. Zymography results of (Figure 2A,B) were analyzed and plotted as shown in (Figure 2C,D). Our data revealed RES-LA concentration-dependent inhibition of MMP-9 with a monophasic dose–response relationship with RES-LA (Figure 2D). Here, RES-LA exhibited an inhibitory activity of MMP-9 up to a concentration 5 µM.

### MTT cytotoxicity assay of RES-LA

**Figure 3:**
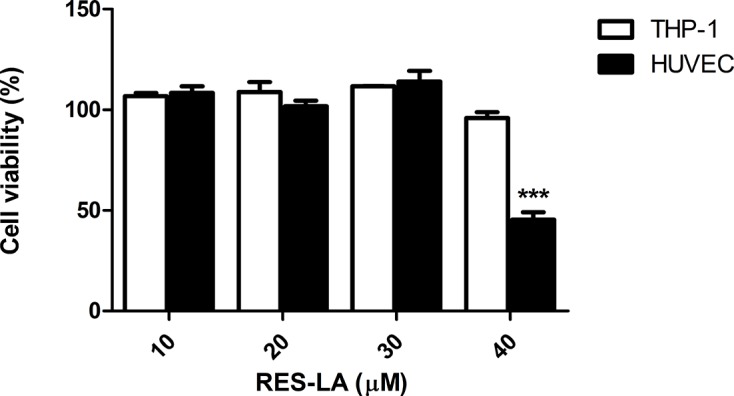
MTT cell viability assay of RES-LA MTT cell viability assay was carried out by seeding THP-1 or HUVEC (10^4^ cells/well) in 96-well plates in the presence of different concentration of RES-LA (10, 20, 40, and 80 µM). Data were normalized using cells incubated with culture medium supplemented with 0.1% NADES, which was considered as the negative control or 1% SDS in 0.01 N HCl as the positive control. Data represent mean ± S.D. ***P≤0.001.

The cytotoxicity of the RES-LA was assessed on primary HUVEC and THP-1 monocytes using the MTT assay (Figure 3). Results showed a total absence of toxicity on THP-1 monocytes was observed up to 80 µM of RES-LA. While a significant reduction in HUVEC by 56% was observed at 40 µM of RES-LA.

### Endothelial permeability assays

To assess the capability of RES-LA to counteract the exacerbation of TNF-α-induced endothelial permeability, three complementary experimental approaches were used in the present study: (i) the endothelial permeability using a FITC-dextran fluorescence spectrometric assay; (ii) the integrity of the intercellular junction protein PECAM-1 and intercellular spaces in HUVEC monolayer by microscopic examination; and (iii) the endothelial permeability using a real-time monitoring of TEER approach.

**Figure 4:**
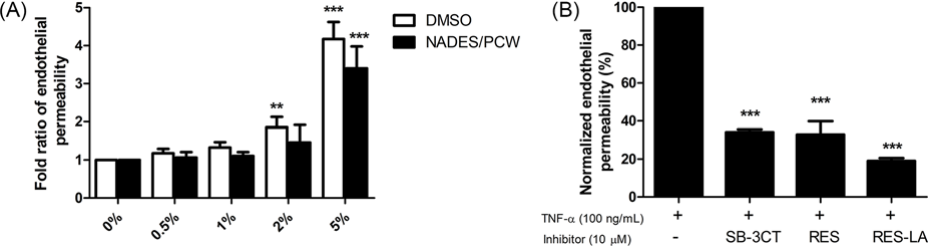
*In vitro* endothelial permeability assay After seeding primary HUVEC (3 × 10^5^) on collagen-coated insert, cells were incubated for 48 h. Different concentrations of NADES or DMSO incubated for 24 h at 37°C with primary HUVEC monolayers (**A**). Cells were incubated with TNF-α 100 ng/ml with or without 10 µM of RES, RES-LA, or SB-3CT (**B**). Data represent mean ± S.D.; **P≤0.01, ***P≤0.001.

First, the effects of different concentrations of NADES or DMSO (0.5, 1, 2, and 5%) were assessed using the HUVEC monolayer permeability exacerbation model and was monitored using FITC-dextran assay (Figure 4A). Results showed that NADES did not induce the endothelial permeability for concentrations below 2%, while DMSO did not exhibit an effect on permeability for concentrations below 1%. The permeability effect of TNF-α alone on HUVEC monolayer was assessed by fluorescent spectrophotometry, our findings showed a clear TNF-α-dependent endothelial permeability enhancement (Figure 4B). In contrast, the incubation of TNF-α-activated HUVEC monolayer in the presence of either SB-3CT, RES, or RES-LA, permitted to observe a marked reduction in the exacerbated endothelial permeability level, getting back to the basal level. Respectively, RES-LA, RES, and SB-3CT were able to reduce the TNF-α-induced permeability respectively by 81, 67, and 63% (Figure 4B).

**Figure 5:**
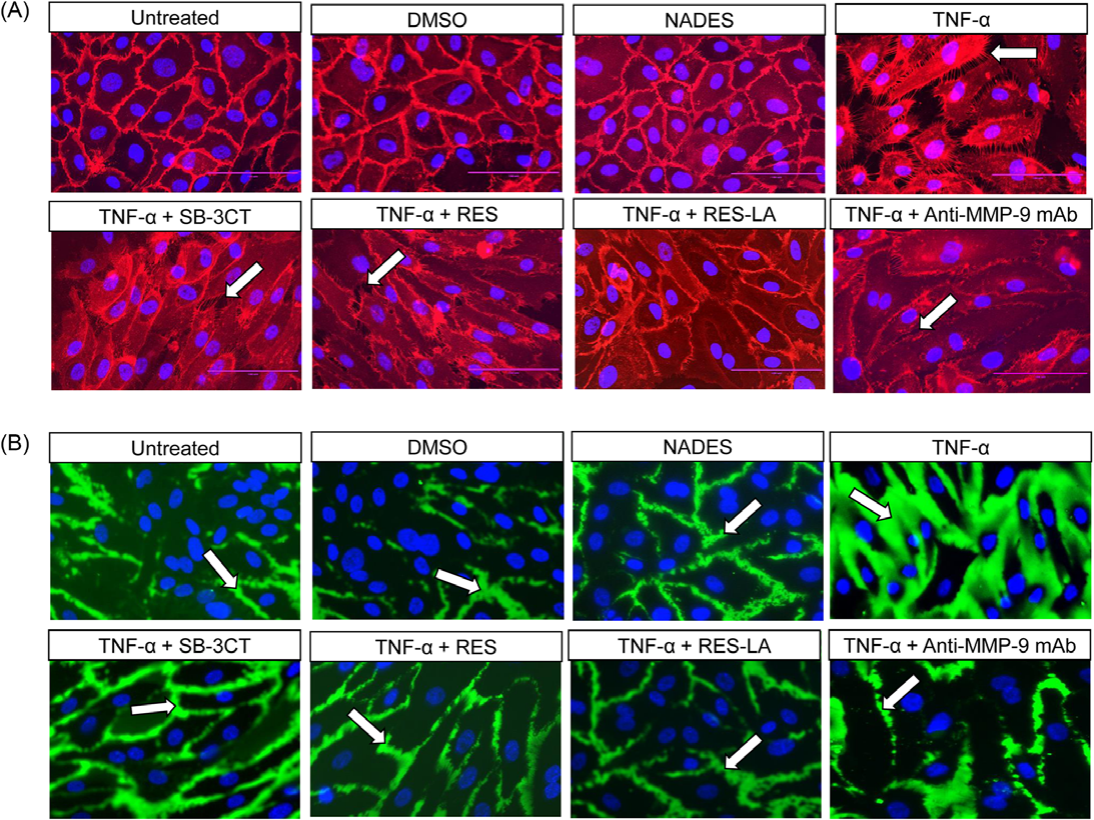
Localization of PECAM-1 and intercellular spaces HUVEC (3 × 10^5^ cells/well) were plated for 48 h on biotinylated-gelatin coated chambers. TNF-α (100 ng/ml) was added in the presence or in the absence of 10 µM RES, RES-LA, SB-3CT, or with anti-MMP-9 mAb for 24 h. All samples were treated either with a Cy3-conjugated mouse anti-human CD31/PECAM-1 mAb (**A**) or with a fluorescein-streptavidin compound (**B**).

In addition, all these inhibitors exhibited the capacity to protect the integrity of the intercellular junction, as illustrated in the HUVEC photomicrographs showing the PECAM-1 endothelial intercellular junction protein integrity (Figure 5A), as well as the biotinylated gelatin representing the intercellular spaces (Figure 5B). To assess whether these observed monolayer disturbances were specifically due to MMP-9, the HUVEC monolayers were incubated with both 100 ng/ml TNF-α and a mouse anti-MMP-9 mAb (Figure 5). The results demonstrated a preservation of both endothelial monolayer and PECAM-1 junction protein integrities.

**Figure 6:**
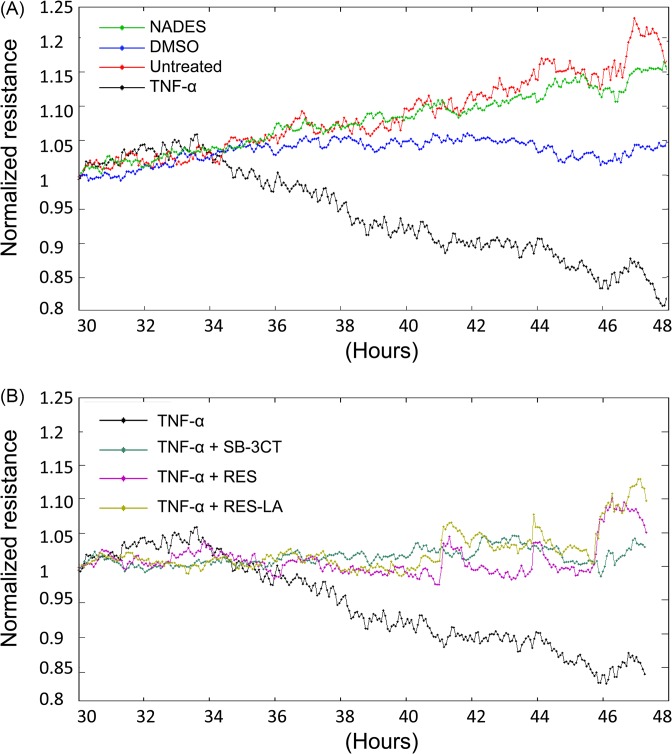
Real-time monitoring of TEER HUVEC were seeded (2 × 10^5^ cells/well) in gelatin A-coated golden ECIS ‘8W10E+’ well arrays. After formation of a confluent HUVEC monolayer, cells were treated with 100 ng/ml TNF-α in the presence or absence of 10 µM of either RES, RES-LA, or SB-3CT. Real-time TEER measurements were done at and 8-s frequency. Results were analyzed using ECIS software. (**A**) Represents untreated, negative controls (0.1% NADES or DMSO), and TNF-α groups. (**B**) Represents RES, RES-LA, or SB-3CT groups.

The real-time monitoring of TEER (Figure 6) showed that TNF-α triggered a decrease in TEER values of the HUVEC confluent cell monolayer, as compared with both untreated and negative control groups, only 4 h (t = 34 h) after activation (Figure 6A). This TNF-α-induced TEER reduction lasted until the end of the experiment, 18 h post-activation of the HUVEC monolayer (t = 48 h). In contrast, RES, RES-LA and SB-3CT reduced the TNF-α-induced resistance declination (Figure 6B). This important observation indicates that the use of MMP-9 inhibitors could preserve the endothelial monolayer integrity.

### Quantitative real-time qPCR to assess *MMP-9* mRNA expression in the activated THP-1 monocytes

**Figure 7:**
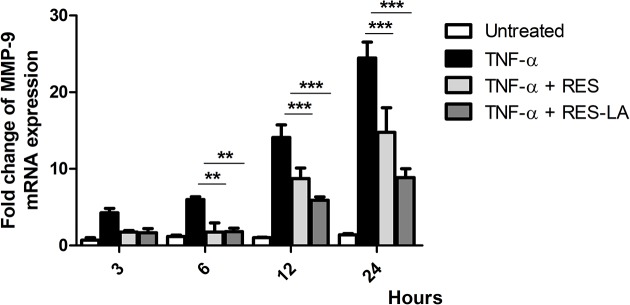
*MMP-9* mRNA expression in RES- and RES-LA-treated monocytes Different groups of TNF-α-activated THP-1 monocytes (7.5 × 10^5^ cells/well) were treated with either 10 µM RES or RES-LA for either 3, 6, 12, or 24 h. Relative quantities of MMP-9 compared with the housekeeping *HMBS* gene expression were assessed using a comparative threshold cycle number (*C*_T_) yield. Data represent the corresponding means ± S.D. **P≤0.01, ***P≤0.001.

The effect of both RES and RES-LA on the *MMP-9* mRNA expression was monitored at 3, 6, 12, and 24 h in TNF-α-activated THP-1 monocytes (Figure 7). Negative vehicle control group was the cell cultivation with 0.1% NADES. Results showed that *MMP-9* mRNA levels were significantly higher in TNF-α-treated THP-1 monocytes as compared with the negative control. Both RES and RES-LA compounds significantly decreased the TNF-α-induced *MMP-9* mRNA expression in a time-dependent manner (Figure 7).

### Phospho-kinase assay of RES and RES-LA in activated THP-1 monocytes

**Figure 8:**
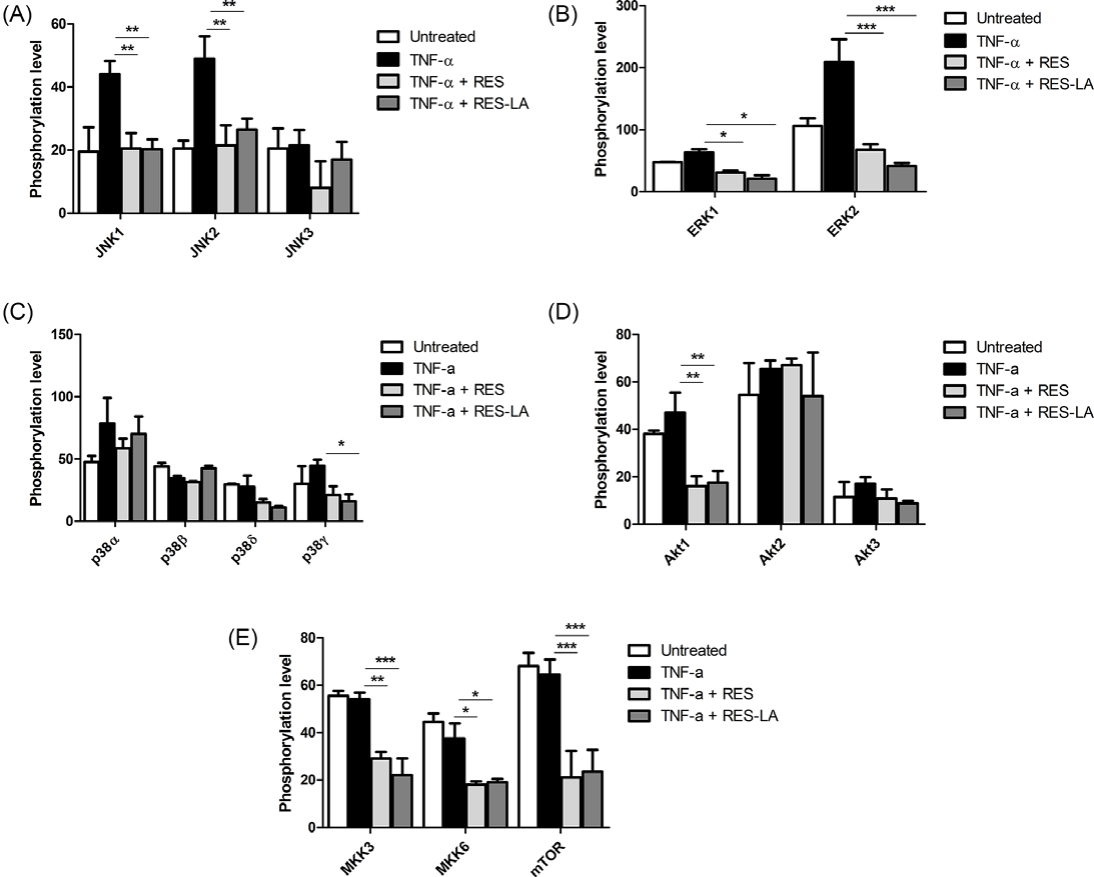
Phospho-kinase status of ERK, JNK, Akt, MKK, and mTOR in response to RES or RES-LA THP-1 monocytes (5 × 10^5^ cells/well) were pre-treated or not with 10 µM of RES or RES-LA for 1 h, followed by 5-min incubation with TNF-α 10 ng/ml. The phosphorylation status of MAP kinases JNK1/2 (**A**), ERK1/2 (**B**), p38 (**C**), as well as Akt1 (**D**), MKK3/6 and mTOR (**E**) kinases were monitored. Data represent mean ± S.D. *P≤0.05, **P≤0.01, ***P≤0.001.

In cancer research, TNF-α was reported to play a role in the progression of cancer by up-regulating the expression of MMP-9 via the activation of ERK1/2, p38, and JNK intracellular signaling pathways, [15,16]. Nevertheless, MEK has been reported to increase cancer progression via MKK3/6-p38 dependent pathways which maintain cancer viability and aggressiveness [17]. For these reasons, we assessed the effect of RES-LA on the phosphorylation level of different cell kinases including MAPK (p38, ERK, JNK), Akt, MKK, and mTOR in TNF-α-activated THP-1 monocytes (Figure 8). These graphs were built from immunoblots (Supplementary Figure S2A–D). Results showed a TNF-α-dependent phosphorylation of ERK1/2, JNK1/2, and p38-α MAPK. Whereas the rest of pathways were not activated by TNF-α as compared with untreated groups. Alternatively, RES and RES-LA have significantly inhibited the TNF-α-dependent phosphorylation of JNK1/2 and ERK1/2 (Figure 8A,B), as well as other TNF-α-independent pathways including p38γ (Figure 8C), MKK3/6 (Figure 8D), and mTOR (Figure 8E). Zymogram analyses revealed that the JNK inhibitor, sp600125, at 5 and 15 µM was able to reduce the MMP-9 activity in TNF-α-activated cells (Supplementary Figure SM).

## Discussion

Vascular leakage is always associated with an exacerbated endothelial permeability, which is due to the degradation of intercellular protein junctions, extracellular matrix, and basement membrane by high expression of active MMP-9 as well as pro-inflammatory cytokines and mediators [18]. Even though RES has been reported to strongly inhibit MMP-9, its use in clinics remains limited due to its poor bioavailability. Therefore, we developed RES-derived lipophenols to overcome these particular concerns.

The results obtained from the assessment of the MMP-9 inhibitory activity of the RES lipophenols (Figure 2) highlight the importance of the position and structure of the FA to confer to these RES-derived compounds and increased MMP-9 inhibitory activity. Indeed, RES-LA, the omega-6 LA incorporated at the 4′ position (**5b**) led to observe the highest activity as compared with both **5a** and **5c** compounds (Figure 1). Considering a previous study that revealed the interaction between RES and the active site residues of MMP-2 and MMP-9 [19], we suggest that the positions of lipids on the RES scaffold structure could have an impact on the resorcinol moiety and the structure–activity relationship of the RES derivatives. This hypothesis could be supported by the facts that the total topology of the lipophenol would be changed depending not only on the position of the FA but also on its type. Since saturated FAs are much less flexible than PUFA, the difference of the inhibitory activity of the unsaturated DHA (six double bonds) and LA (two double bonds) conjugates suggest that the spatial environment of the lipophenol could have a potential impact on the mechanism of action of those compounds. Afterward, we assessed the cytotoxicity assay of most active and soluble lipophenol derivative, RES-LA, on THP-1 and HUVEC (Figure 3). Our data revealed a less cytotoxic profile of RES-LA as compared with the parent compound RES as assessed in our previous study [14]. Additionally, we chose NADES as a solvent, since it is believed to be less toxic than other organic solvents due to its natural composition [13]. However, in our previous study, the compared cytotoxicity levels between NADES (1,2-propanediol:choline chloride:water, 1:1:1) and DMSO were not different [14]. We also demonstrated in that same study that NADES was able to improve the MMP-9 inhibitory activity of RES by at least ten fold higher than DMSO, thus permitting to evidence the hormetic mode of action of RES.

In the present study, we examined the effect of RES and RES-LA on reducing the exacerbated endothelial permeability induced by TNF-α. The latter is reported to play a major role in inflammatory response and induction of an exacerbated increase in endothelial permeability resulting in a pathological vascular leakage [18]. Our data revealed that RES and RES-LA were not only capable of decreasing the *in vitro* endothelial permeability as monitored by spectrophotometry (Figure 4) and in real-time using TEER (Figure 6), but also prevented the dissociation of the intercellular adherent junctions including PECAM-1 by inhibiting MMP-9 activity (Figure 5A). The specificity of MMP-9-induced permeability was validated by using anti-MMP-9 mAb that reduced the degradation of PECAM-1 as shown in (Figure 5A). These findings are consistent with Kato and colleagues [20], who studied the relation between MMP-9 deficiency and PECAM-1 in injured steatotic livers from transgenic MMP-9^−/−^ mice.

To assess the cellular pathways by which RES-LA inhibits MMP-9 expression (Figure 7), we evaluated the phosphorylation status of different kinases including MAP-kinases in activated monocytes (Figure 8). Amongst the tested pathways, we found that TNF-α activates ERK1/2 and JNK1/2 signaling pathways in THP-1 monocytes. These pathways were deactivated by either RES or RES-LA (Figure 8A,B). Indeed, ERK-dependent TNF-α phosphorylation has been reported to induce MMP-9 expression [21,22]. Yet, JNK-dependent MMP-9 activation remains controversial depending on the experimental conditions [16,22]. Therefore, we validated our results by using JNK inhibitor (sp00125) in zymogram analysis that has shown to decrease MMP-9 activity (Supplementary Figure S3). Alternatively, some pathways (p38γ, MKK3/6, Akt1, and mTOR) were not activated by TNF-α in THP-1 monocytes. These pathways were also inhibited by either RES or RES-LA. Therefore, it is interesting to stress that RES has been reported to promote autophagy and induce its anti-inflammatory effect by deactivating mTOR signaling pathway [23,24]. In addition, RES was also shown to prevent TNF-α-induced muscle atrophy via the Akt/mTOR-dependent pathway [25]. In view of RES-LA regulates the same signaling pathways as the parent molecule, RES, we hypothesize that it could exert the same anti-inflammatory and anticancer activities as the parent molecule, RES.

## Conclusion

Altogether the results of the present study clearly show that RES-LA exhibited in our study a drastic reduction in the exacerbated pathological endothelial permeability by inhibiting the activity and expression of MMP-9 and consequently preserving the endothelial intercellular junction integrity. Moreover, this work has also evidenced that in THP-1 monocytes, RES-LA was able to inhibit the TNF-α-induced MMP-9 expression by deactivating ERK1/2 and JNK1/2 MAPK. Besides inhibiting the phosphorylation of other signaling pathways including p38-γ, MKK3/6, Akt1, and mTOR. In addition, the present study provides a novel non-toxic RES derivative, which can overcome the bioavailability limitations of RES. Thus, RES-LA could be considered as a novel promising NADES-formulated compound to preserve the endothelial barrier integrity able to mitigate the inflammatory-exacerbated vascular endothelial dysfunction. Obviously, these *in vitro* results need to be confirmed and extended using appropriate animal models to prepare pre-clinical trials.

## Supporting information

**supplementary Figures:** 

## Abbreviations

Akt: Protein Kinase B (PKB)
BE: behenic acid
CD31: cluster of diffrenciation 31
Cy3: cyanin 3
DCC/DMAP: dicyclohexylcardodiimide and dimethylamino pyridine
DHA: docosahexaenoic acid
ERK1/2: extracellular signal regulated kinases 1/2
FA: fatty acid
HUVEC: human umbilical vein endothelial cells
HMBS: hydroxymethylbilane synthase
JNK 1/2: c-jun N-terminal kinases 1./2
LA: linoleic acid
mAb: monoclonal antibody
MAPK: mitogen-activatedprotein kinase
MKK: mitogen-activated protein kinase kinase
MMP: matrix metalloproteinase
mTOR: mammalian target of rapamycin
NADES: natural deep eutectic solvent
PCW: 1,2-propanediol:choline chloride:water mix
PECAM-1: platelet endothelial cell adhesion molecule-1
PUFA: polyunsaturated fatty acid
qPCR: quantitative polymerase chain reaction
RES: resveratrol
RPMI-1640: Roswell Park Memorial Institute (1640)
TEER: trans-endothelial electric resistance
THP1: monocytic cell line
TIPS: triisopropyl silyl
TNF: tomor necrosis factor
SB-3CT: 2-(((4-Phenoxyphenyl)sulfonyl)methym)-Thiirane
