# Supplementary material for: Resveratrol-Linoleate protects from exacerbated endothelial permeability via a drastic inhibition of the MMP-9 activity
Source: Biosci Rep. 2018 Jul 31;38(4):BSR20171712. doi: 10.1042/BSR20171712 (PMC6066651; doi:10.1042/BSR20171712)
Supplement: Supplementary file 1 [file bsr20171712_Supp1.pdf]

NMR Spectra figures

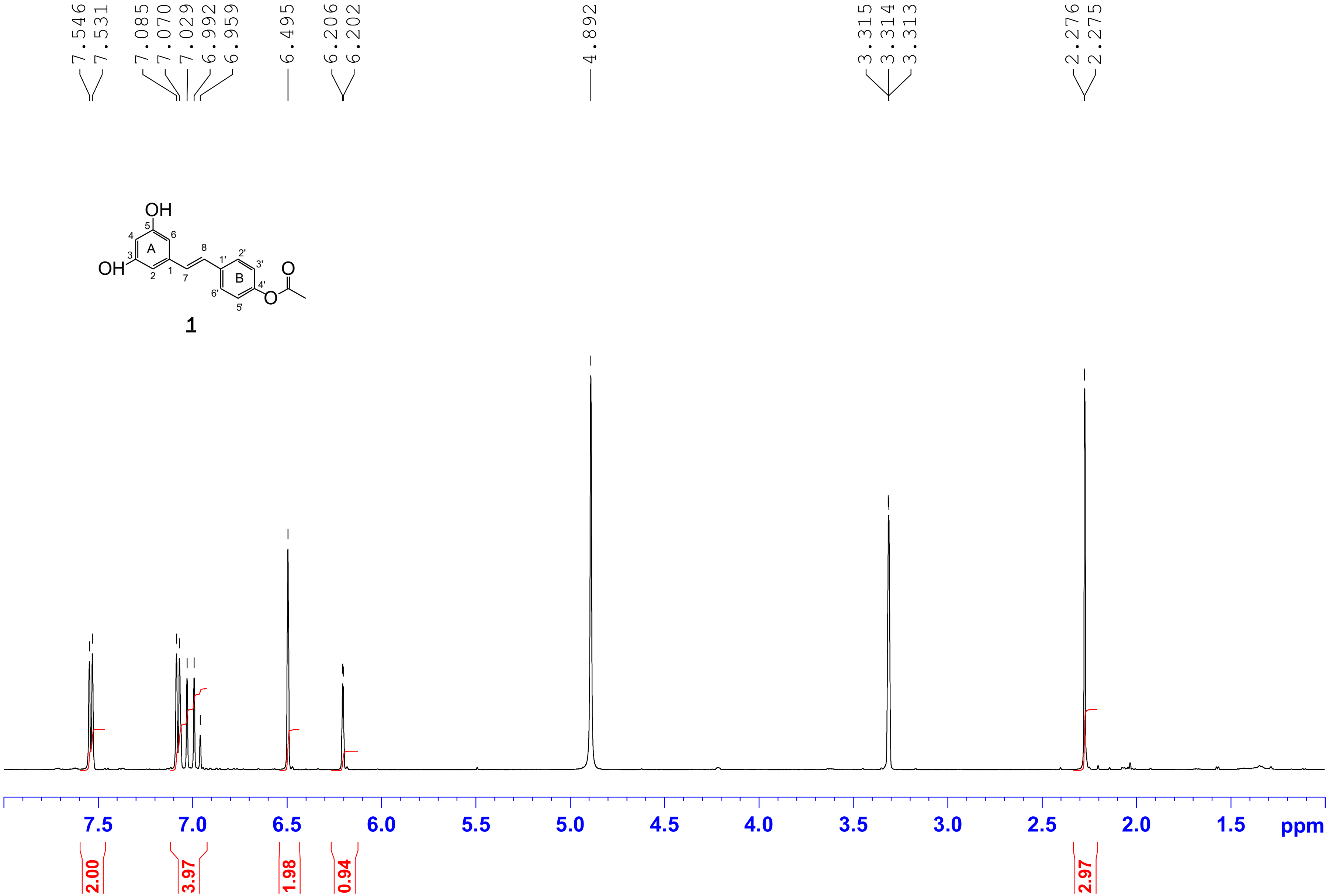
7.546
7.531
7.085
7.070
7.029
6.992
6.959
6.495
6.206
6.202
4.892
3.315
3.314
3.313
2.276
2.275
OH
OH
A
B
O
O
1
7.5
7.0
6.5
6.0
5.5
5.0
4.5
4.0
3.5
3.0
2.5
2.0
1.5
ppm
2.00
3.97
1.98
0.94
2.97

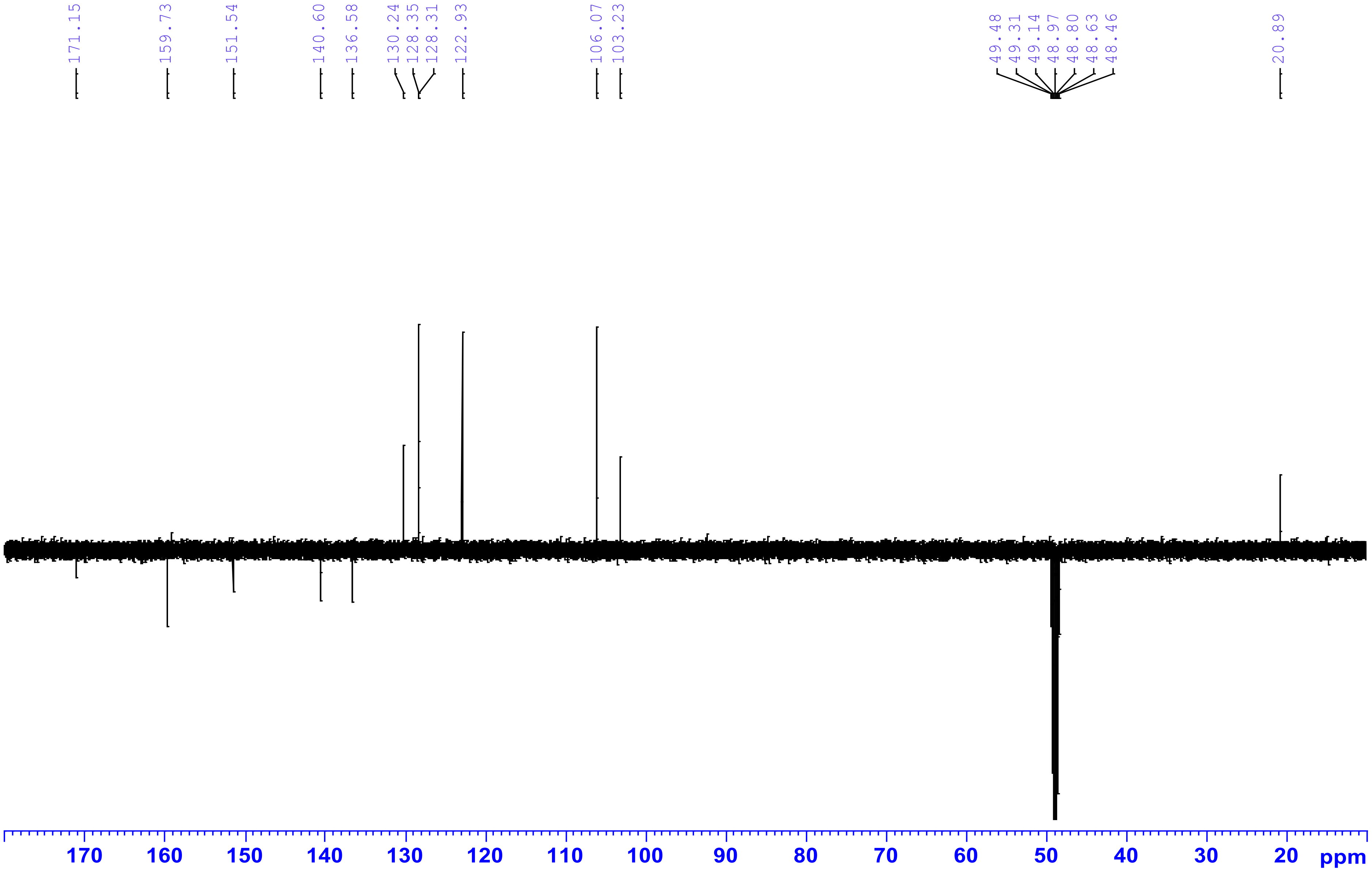
171.15
159.73
151.54
140.60
136.58
130.24
128.35
128.31
122.93
106.07
103.23
49.48
49.31
49.14
48.97
48.80
48.63
48.46
20.89
170
160
150
140
130
120
110
100
90
80
70
60
50
40
30
20
ppm

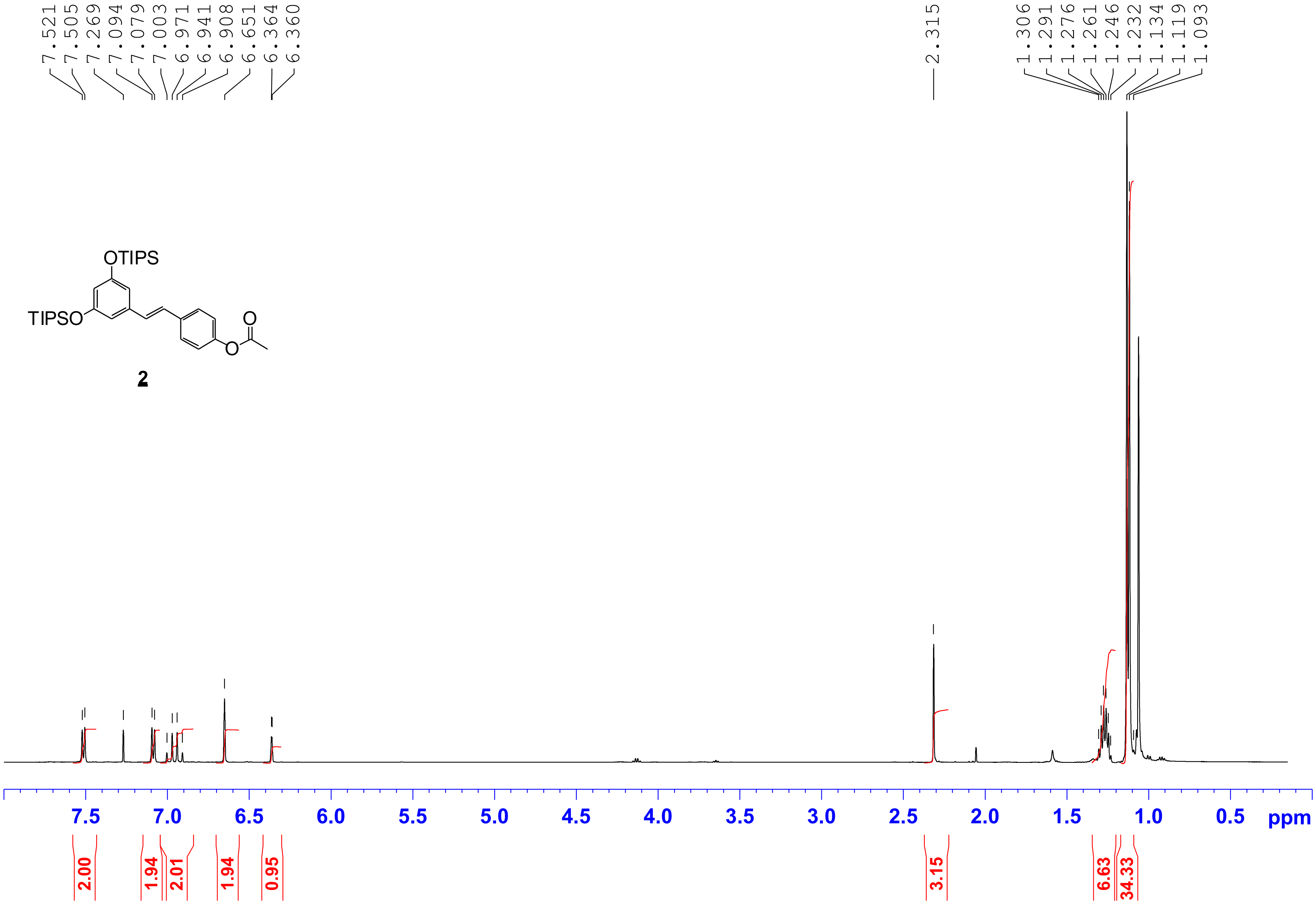

7.521
7.505
7.269
7.094
7.079
7.003
6.971
6.941
6.908
6.651
6.364
6.360
2.315
1.306
1.291
1.276
1.261
1.246
1.232
1.134
1.119
1.093
OTIPS
TIPSO
O
O
2
7.5
7.0
6.5
6.0
5.5
5.0
4.5
4.0
3.5
3.0
2.5
2.0
1.5
1.0
0.5
ppm
2.00
1.94
2.01
1.94
0.95
3.15
6.63
34.33

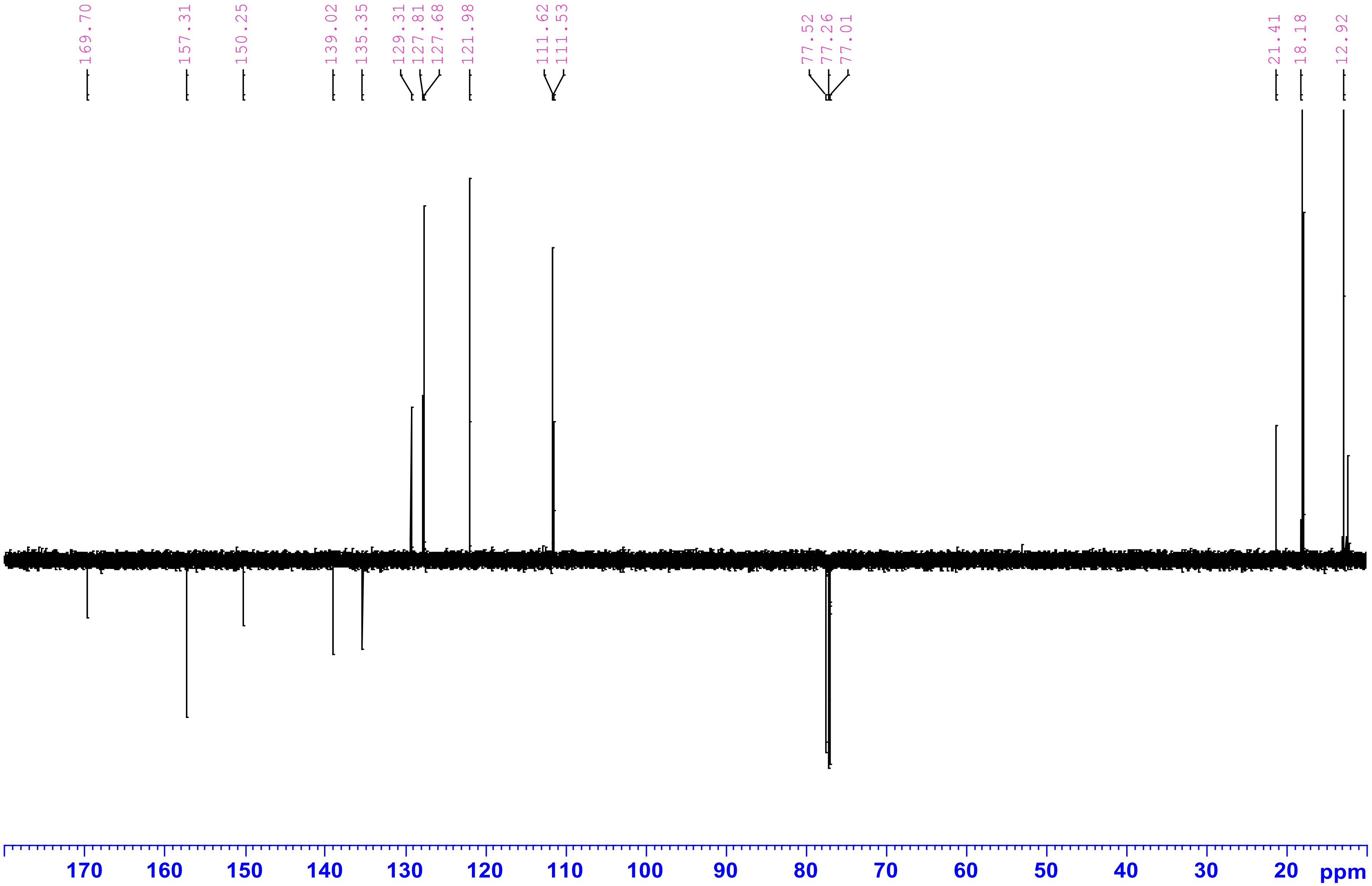
169.70
157.31
150.25
139.02
135.35
129.31
127.81
127.68
121.98
111.62
111.53
77.52
77.26
77.01
21.41
18.18
12.92
170
160
150
140
130
120
110
100
90
80
70
60
50
40
30
20
ppm

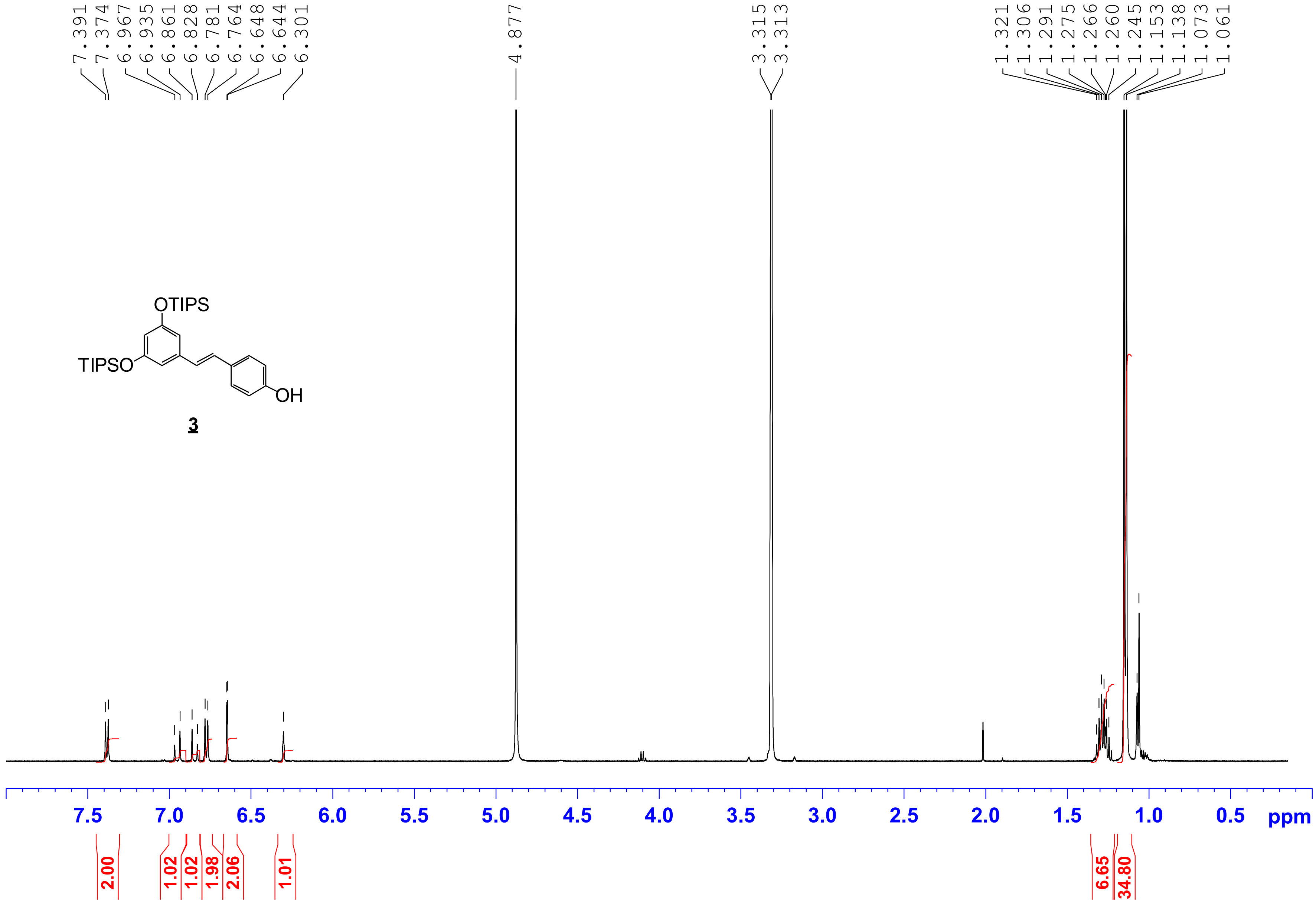
7.391
7.374
6.967
6.935
6.861
6.828
6.781
6.764
6.648
6.644
6.301
4.877
3.315
3.313
1.321
1.306
1.291
1.275
1.266
1.260
1.245
1.153
1.138
1.073
1.061
OTIPS
TIPSO
OH
3
7.5
7.0
6.5
6.0
5.5
5.0
4.5
4.0
3.5
3.0
2.5
2.0
1.5
1.0
0.5
ppm
2.00
1.02
1.02
1.98
2.06
1.01
6.65
34.80

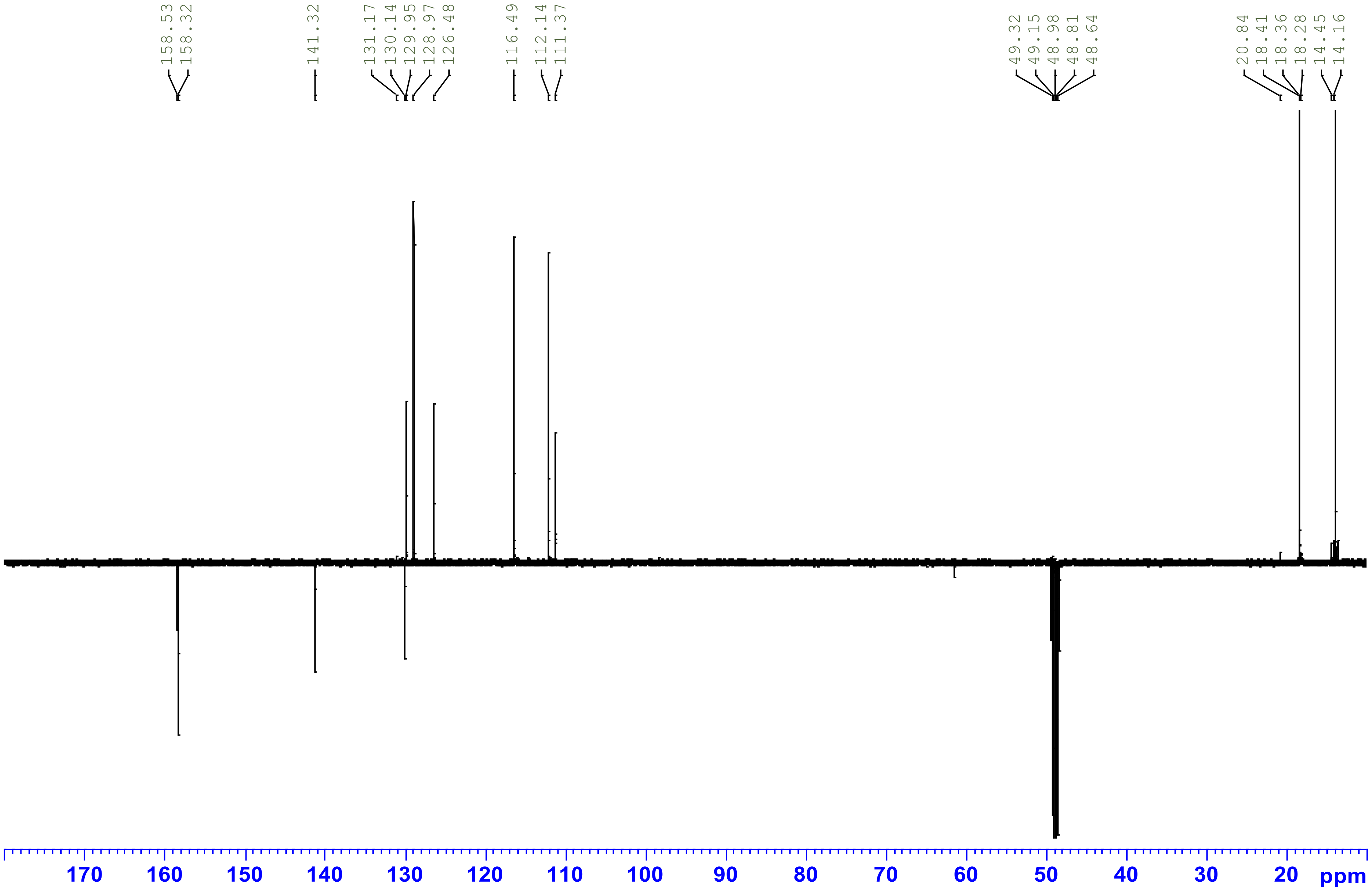
158.53
158.32
141.32
131.17
130.14
129.95
128.97
126.48
116.49
112.14
111.37
49.32
49.15
48.98
48.81
48.64
20.84
18.41
18.36
18.28
14.45
14.16
170
160
150
140
130
120
110
100
90
80
70
60
50
40
30
20
ppm

4a Resv-C22-TIPS CEC125F1 H et C CDCl3

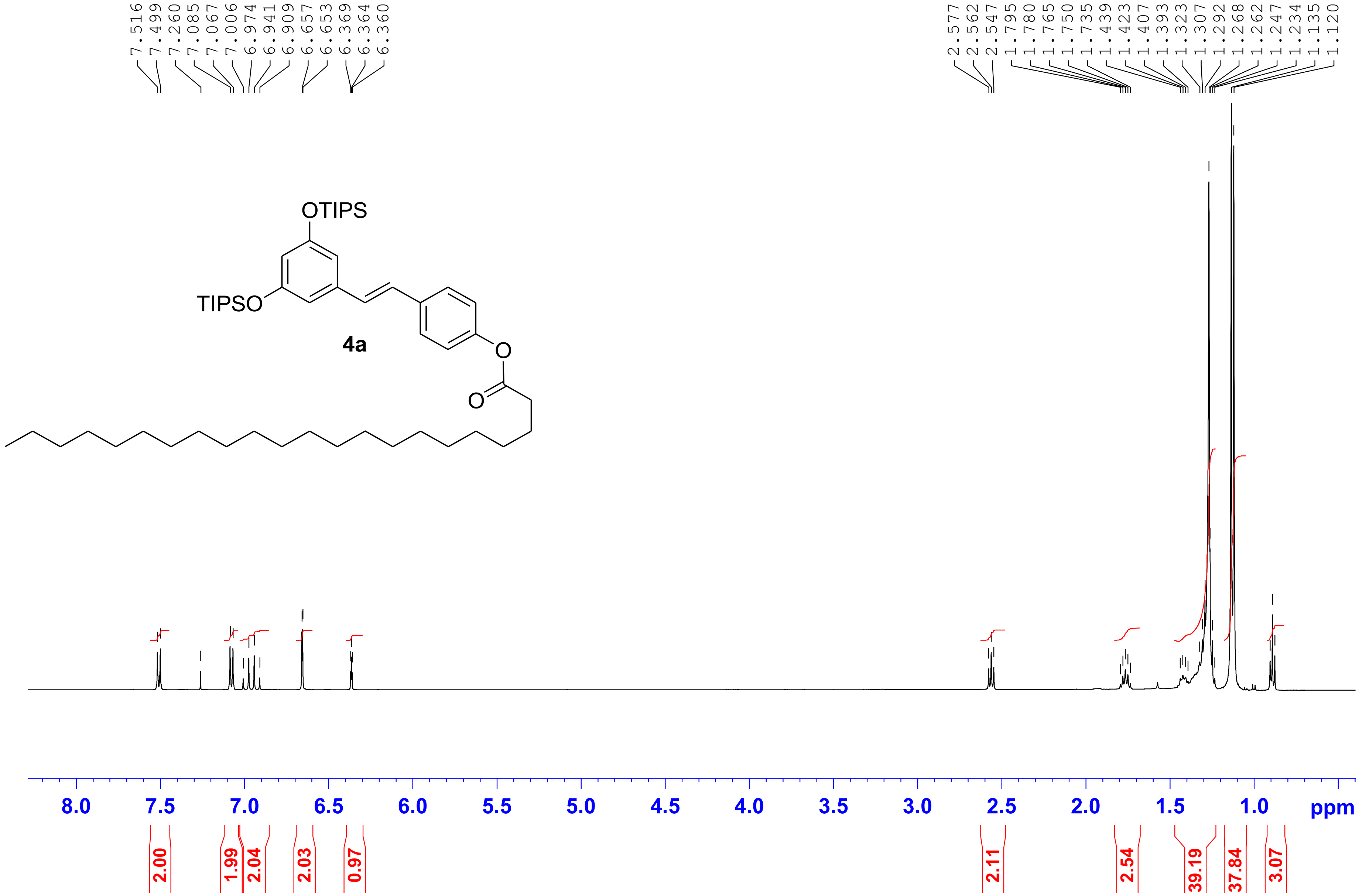
7.516
7.499
7.260
7.085
7.067
7.006
6.974
6.941
6.909
6.657
6.653
6.369
6.364
6.360
2.577
2.562
2.547
1.795
1.780
1.765
1.750
1.735
1.439
1.423
1.407
1.393
1.323
1.307
1.292
1.268
1.262
1.247
1.234
1.135
1.120
OTIPS
TIPSO
O
O
4a
8.0
7.5
7.0
6.5
6.0
5.5
5.0
4.5
4.0
3.5
3.0
2.5
2.0
1.5
1.0
ppm
2.00
1.99
2.04
2.03
0.97
2.11
2.54
39.19
37.84
3.07

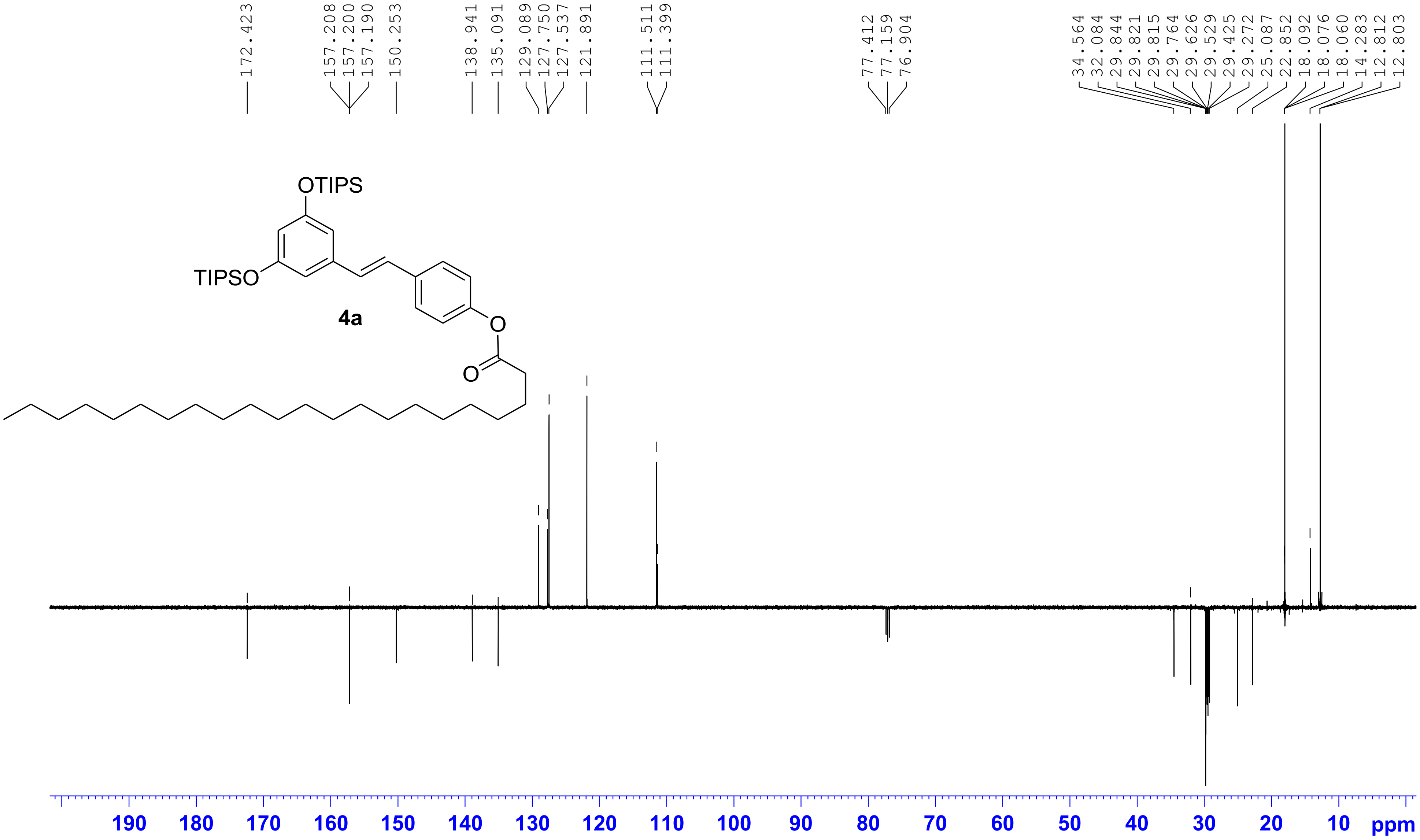
172.423
157.208
157.200
157.190
150.253
138.941
135.091
129.089
127.750
127.537
121.891
111.511
111.399
77.412
77.159
76.904
34.564
32.084
29.844
29.821
29.815
29.764
29.626
29.529
29.425
29.272
25.087
22.852
18.092
18.076
18.060
14.283
12.812
12.803
OTIPS
TIPSO
O
O
4a
190 180 170 160 150 140 130 120 110 100 90 80 70 60 50 40 30 20 10 ppm

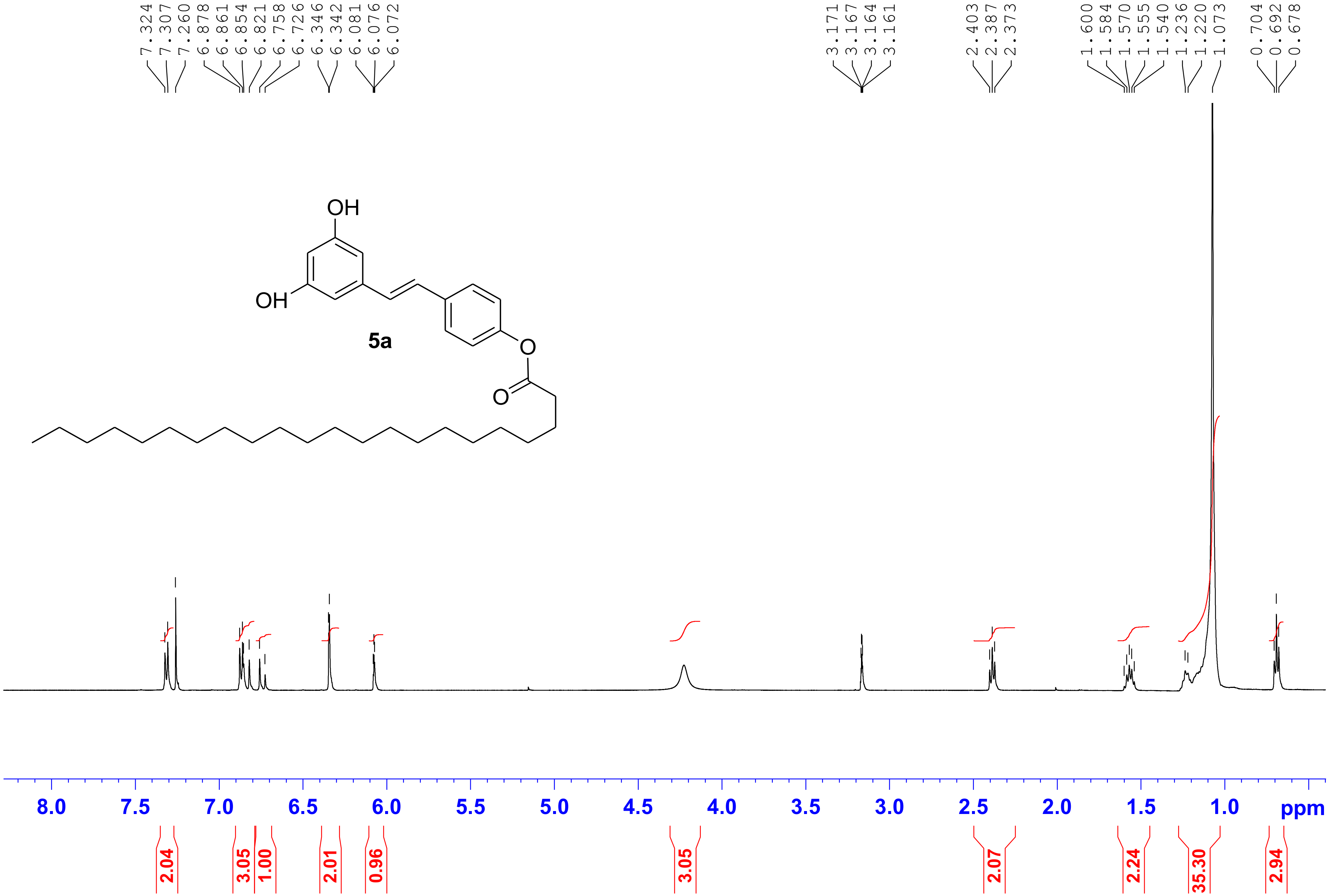

7.324
7.307
7.260
6.878
6.861
6.854
6.821
6.758
6.726
6.346
6.342
6.081
6.076
6.072
3.171
3.167
3.164
3.161
2.403
2.387
2.373
1.600
1.584
1.570
1.555
1.540
1.236
1.220
1.073
0.704
0.692
0.678
OH
OH
5a
O
O
8.0
7.5
7.0
6.5
6.0
5.5
5.0
4.5
4.0
3.5
3.0
2.5
2.0
1.5
1.0
ppm
2.04
3.05
1.00
2.01
0.96
3.05
2.07
2.24
35.30
2.94

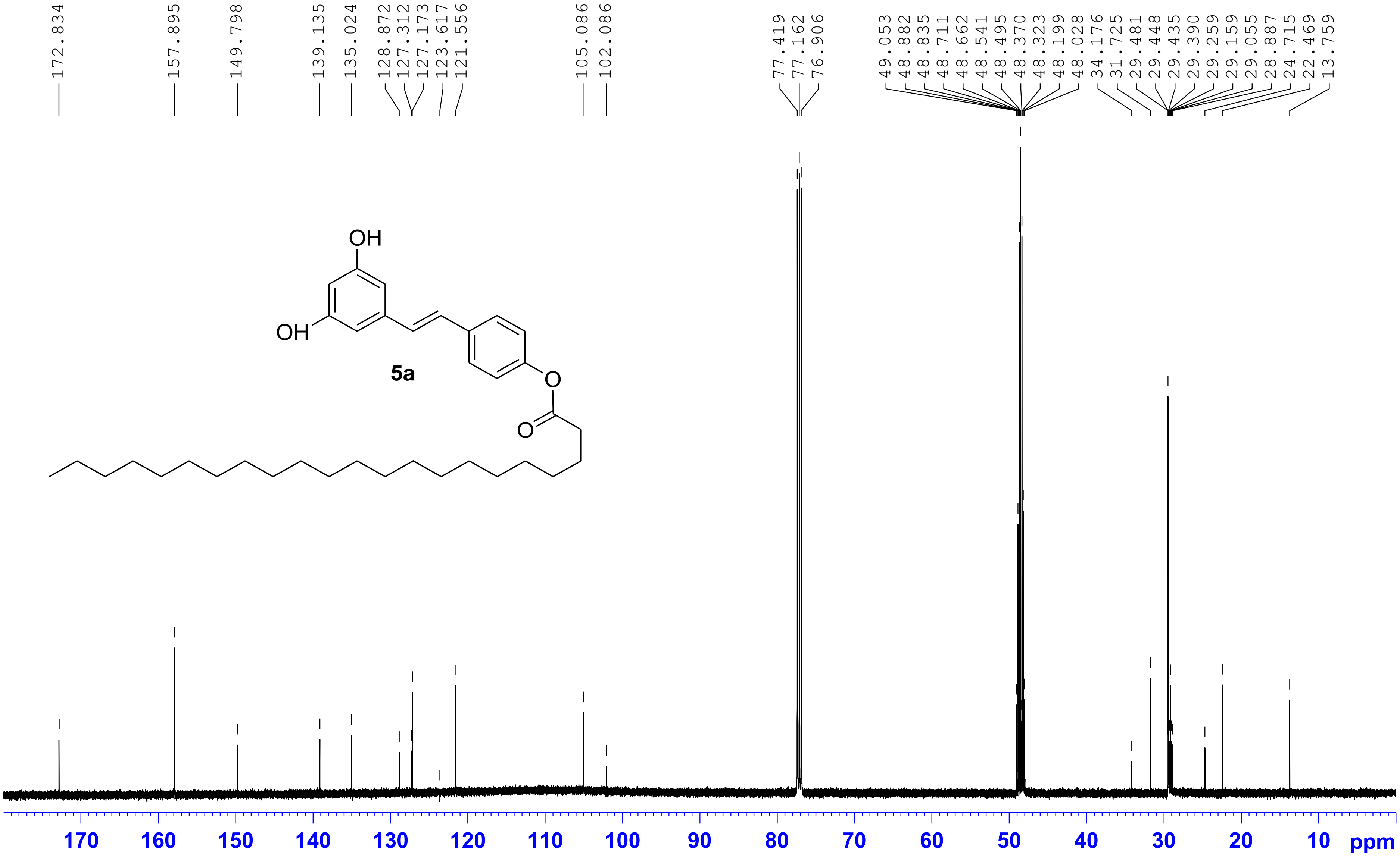
172.834
157.895
149.798
139.135
135.024
128.872
127.312
127.173
123.617
121.556
105.086
102.086
77.419
77.162
76.906
49.053
48.882
48.835
48.711
48.662
48.541
48.495
48.370
48.323
48.199
48.028
34.176
31.725
29.481
29.448
29.435
29.390
29.259
29.159
29.055
28.887
24.715
22.469
13.759
OH
OH
5a
O
O
170
160
150
140
130
120
110
100
90
80
70
60
50
40
30
20
10
ppm

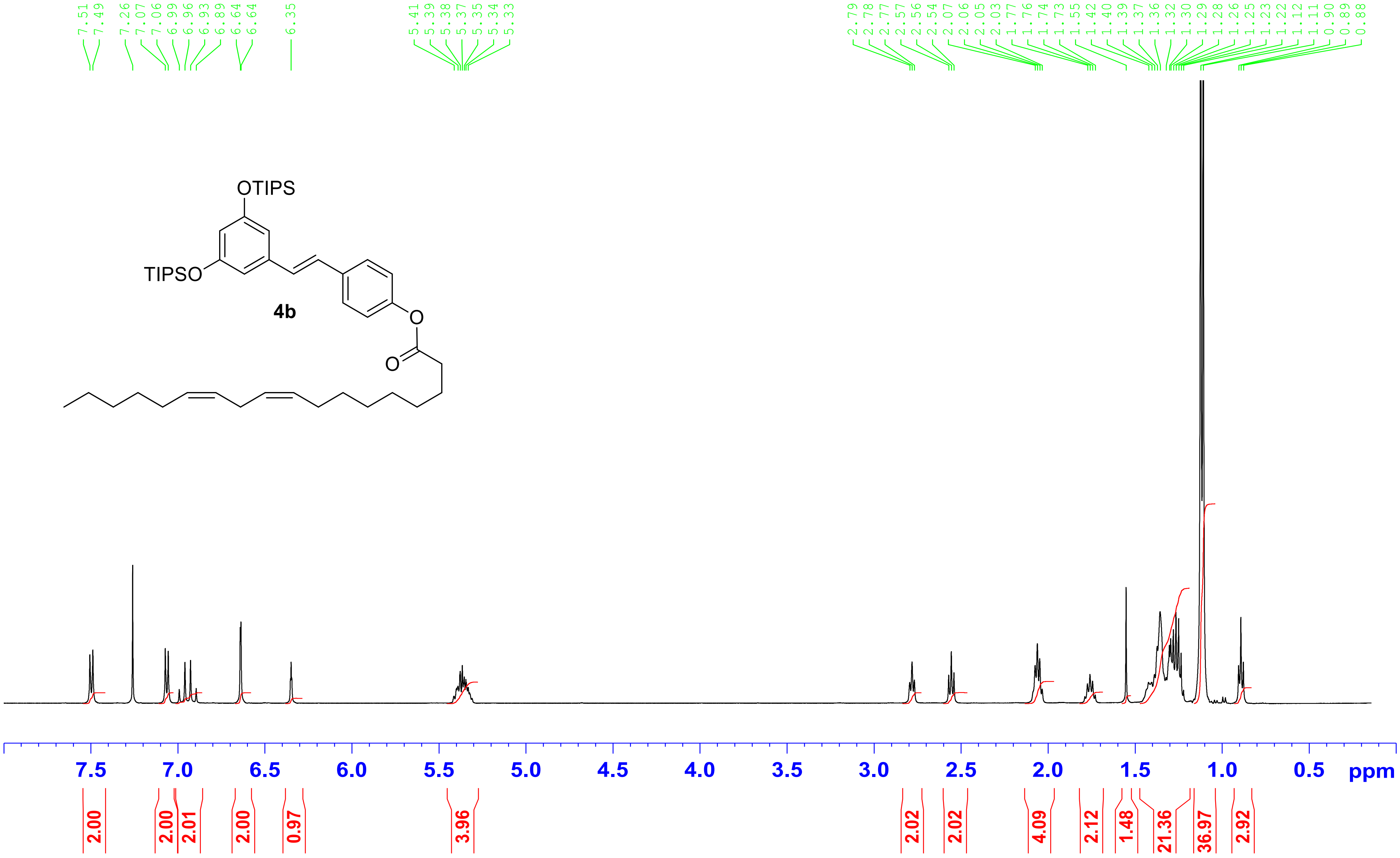

7.51
7.49
7.26
7.07
7.06
6.99
6.96
6.93
6.89
6.64
6.64
6.35
5.41
5.39
5.38
5.37
5.35
5.34
5.33
2.79
2.78
2.77
2.57
2.56
2.54
2.07
2.06
2.05
2.03
1.77
1.76
1.74
1.73
1.55
1.42
1.40
1.39
1.37
1.36
1.32
1.30
1.29
1.28
1.26
1.25
1.23
1.22
1.12
1.11
0.90
0.89
0.88
OTIPS
TIPSO
4b
O
O
7.5
7.0
6.5
6.0
5.5
5.0
4.5
4.0
3.5
3.0
2.5
2.0
1.5
1.0
0.5
ppm
2.00
2.00
2.01
2.00
0.97
3.96
2.02
2.02
4.09
2.12
1.48
21.36
36.97
2.92

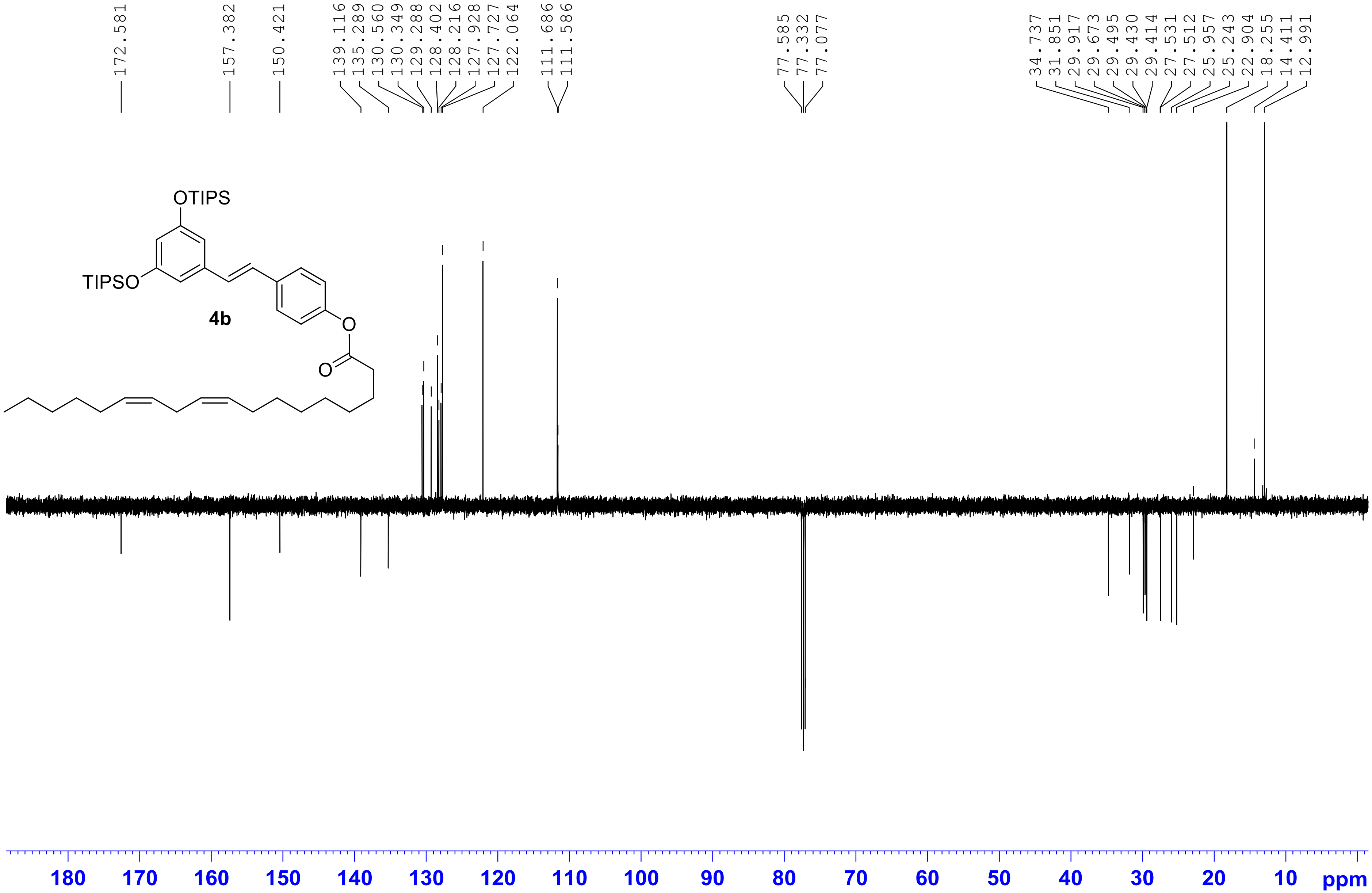

172.581
157.382
150.421
139.116
135.289
130.560
130.349
129.288
128.402
128.216
127.928
127.727
122.064
111.686
111.586
77.585
77.332
77.077
34.737
31.851
29.917
29.673
29.495
29.430
29.414
27.531
27.512
25.957
25.243
22.904
18.255
14.411
12.991
OTIPS
TIPSO
4b
O
O
180
170
160
150
140
130
120
110
100
90
80
70
60
50
40
30
20
10
ppm

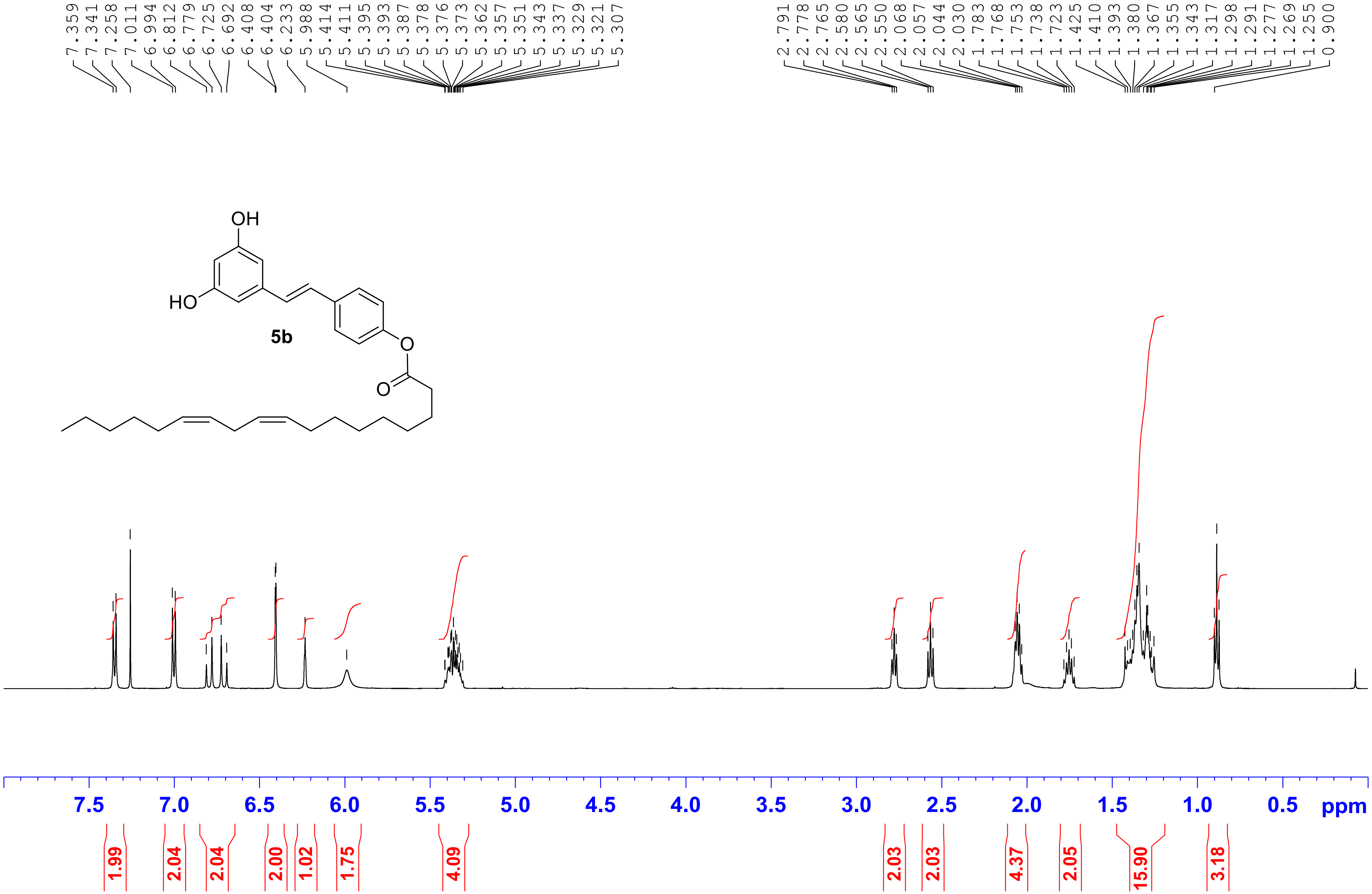
7.359
7.341
7.258
7.011
6.994
6.812
6.779
6.725
6.692
6.408
6.404
6.233
5.988
5.414
5.411
5.395
5.393
5.387
5.378
5.376
5.373
5.362
5.357
5.351
5.343
5.337
5.329
5.321
5.307
2.791
2.778
2.765
2.580
2.565
2.550
2.068
2.057
2.044
2.030
1.783
1.768
1.753
1.738
1.723
1.425
1.410
1.393
1.380
1.367
1.355
1.343
1.317
1.298
1.291
1.277
1.269
1.255
0.900
OH
HO
5b
O
O
7.5
7.0
6.5
6.0
5.5
5.0
4.5
4.0
3.5
3.0
2.5
2.0
1.5
1.0
0.5
ppm
1.99
2.04
2.04
2.00
1.02
1.75
4.09
2.03
2.03
4.37
2.05
15.90
3.18

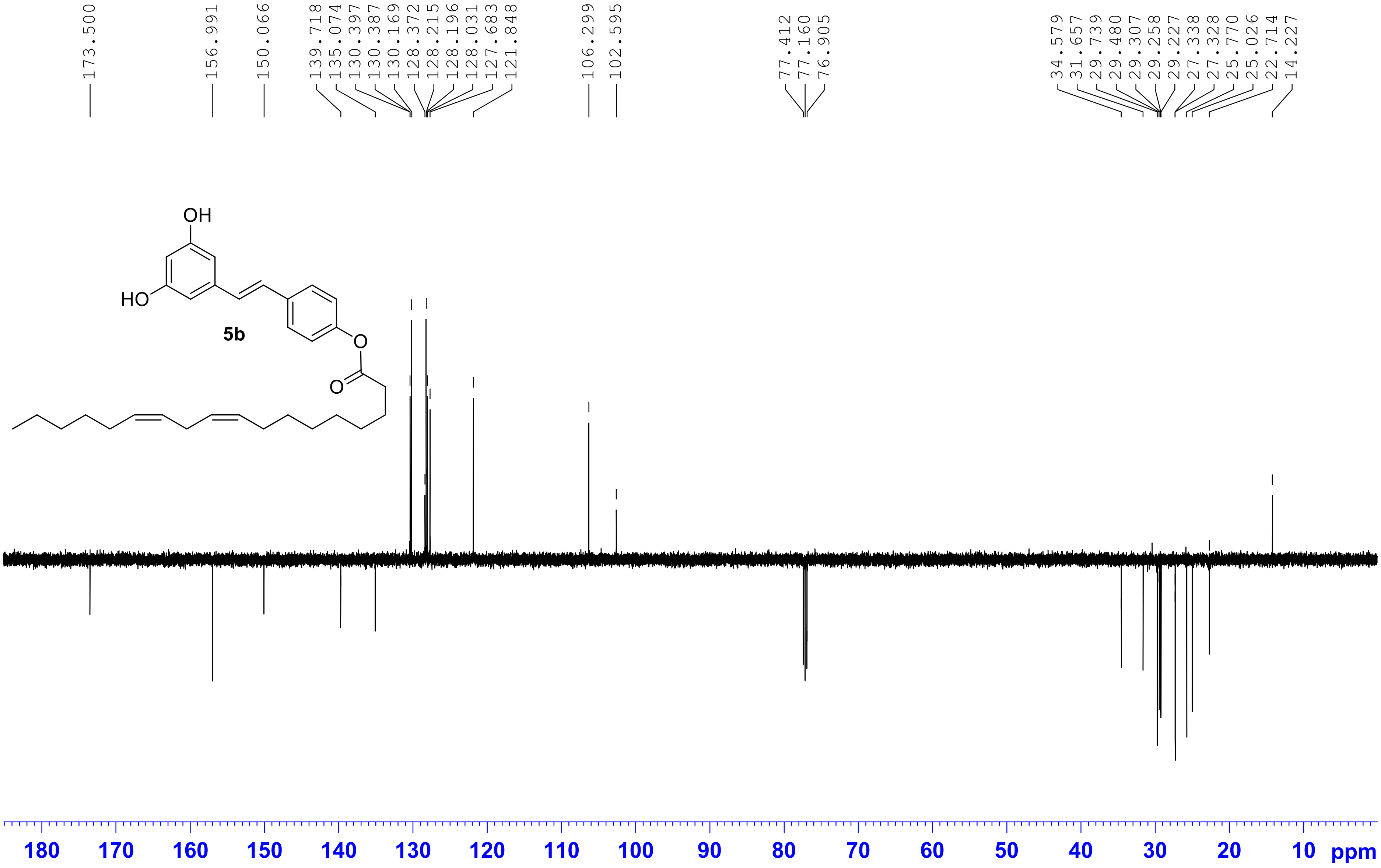

173.500
156.991
150.066
139.718
135.074
130.397
130.387
130.169
128.372
128.215
128.196
128.031
127.683
121.848
106.299
102.595
77.412
77.160
76.905
34.579
31.657
29.739
29.480
29.307
29.258
29.227
27.338
27.328
25.770
25.026
22.714
14.227
OH
HO
O
O
5b
180
170
160
150
140
130
120
110
100
90
80
70
60
50
40
30
20
10
ppm

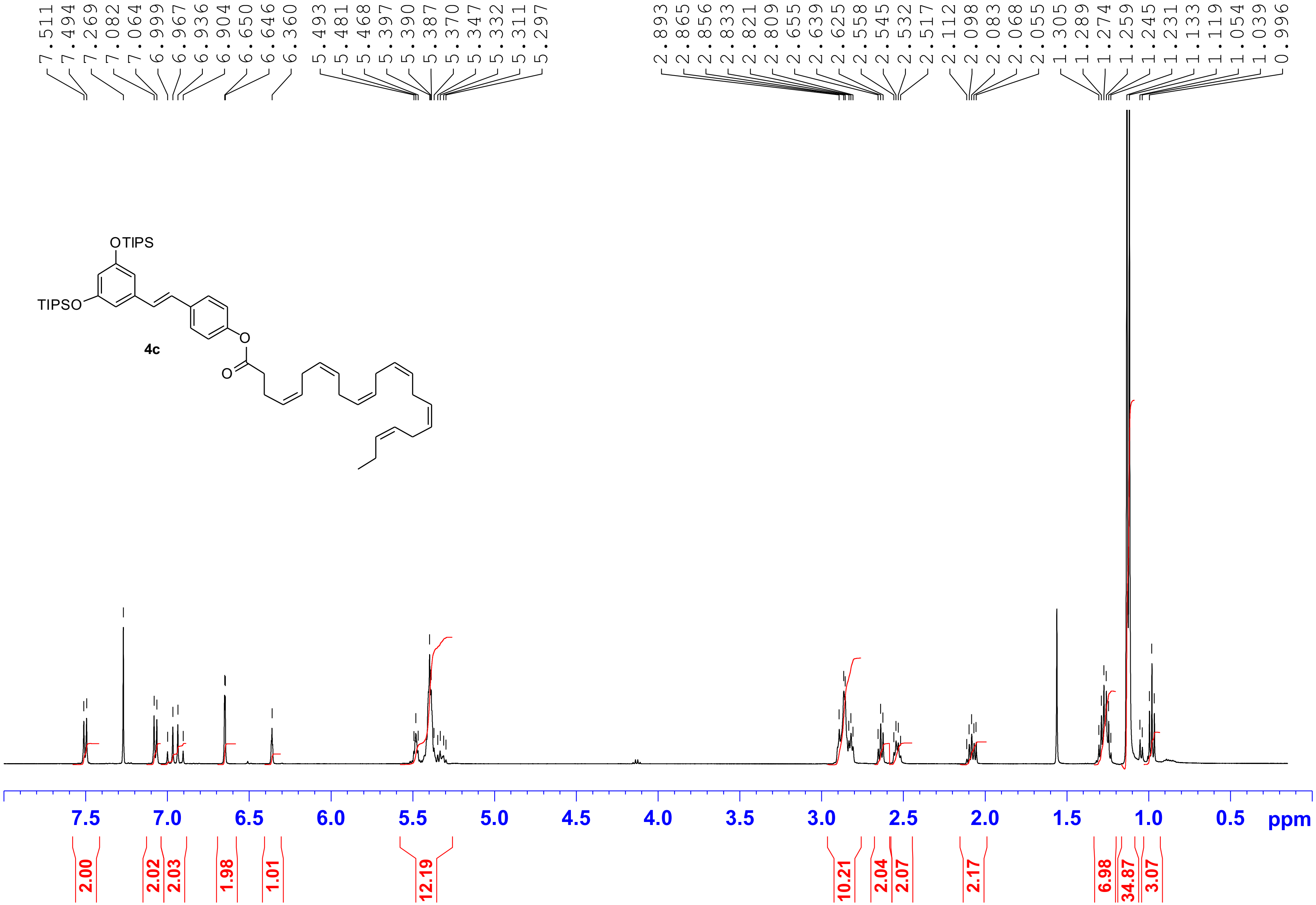
7.511
7.494
7.269
7.082
7.064
6.999
6.967
6.936
6.904
6.650
6.646
6.360
5.493
5.481
5.468
5.397
5.390
5.387
5.370
5.347
5.332
5.311
5.297
2.893
2.865
2.856
2.833
2.821
2.809
2.655
2.639
2.625
2.558
2.545
2.532
2.517
2.112
2.098
2.083
2.068
2.055
1.305
1.289
1.274
1.259
1.245
1.231
1.133
1.119
1.054
1.039
0.996
OTIPS
TIPSO
O
O
4c
7.5
7.0
6.5
6.0
5.5
5.0
4.5
4.0
3.5
3.0
2.5
2.0
1.5
1.0
0.5
ppm
2.00
2.02
2.03
1.98
1.01
12.19
10.21
2.04
2.07
2.17
6.98
34.87
3.07

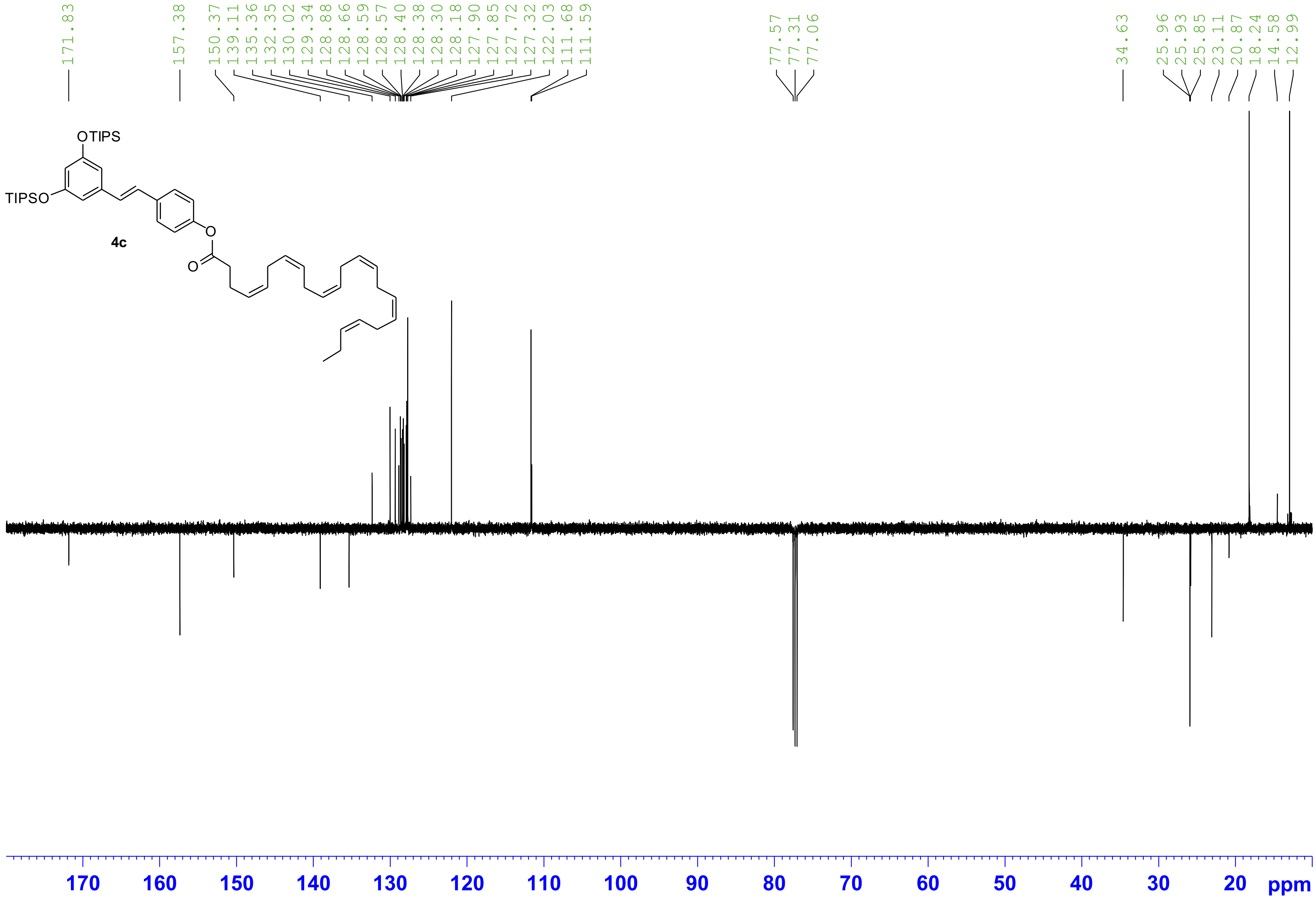
171.83
157.38
150.37
139.11
135.36
132.35
130.02
129.34
128.88
128.66
128.59
128.57
128.40
128.38
128.30
128.18
127.90
127.85
127.72
127.32
122.03
111.68
111.59
77.57
77.31
77.06
34.63
25.96
25.93
25.85
23.11
20.87
18.24
14.58
12.99
OTIPS
TIPSO
O
O
4c
170
160
150
140
130
120
110
100
90
80
70
60
50
40
30
20
ppm

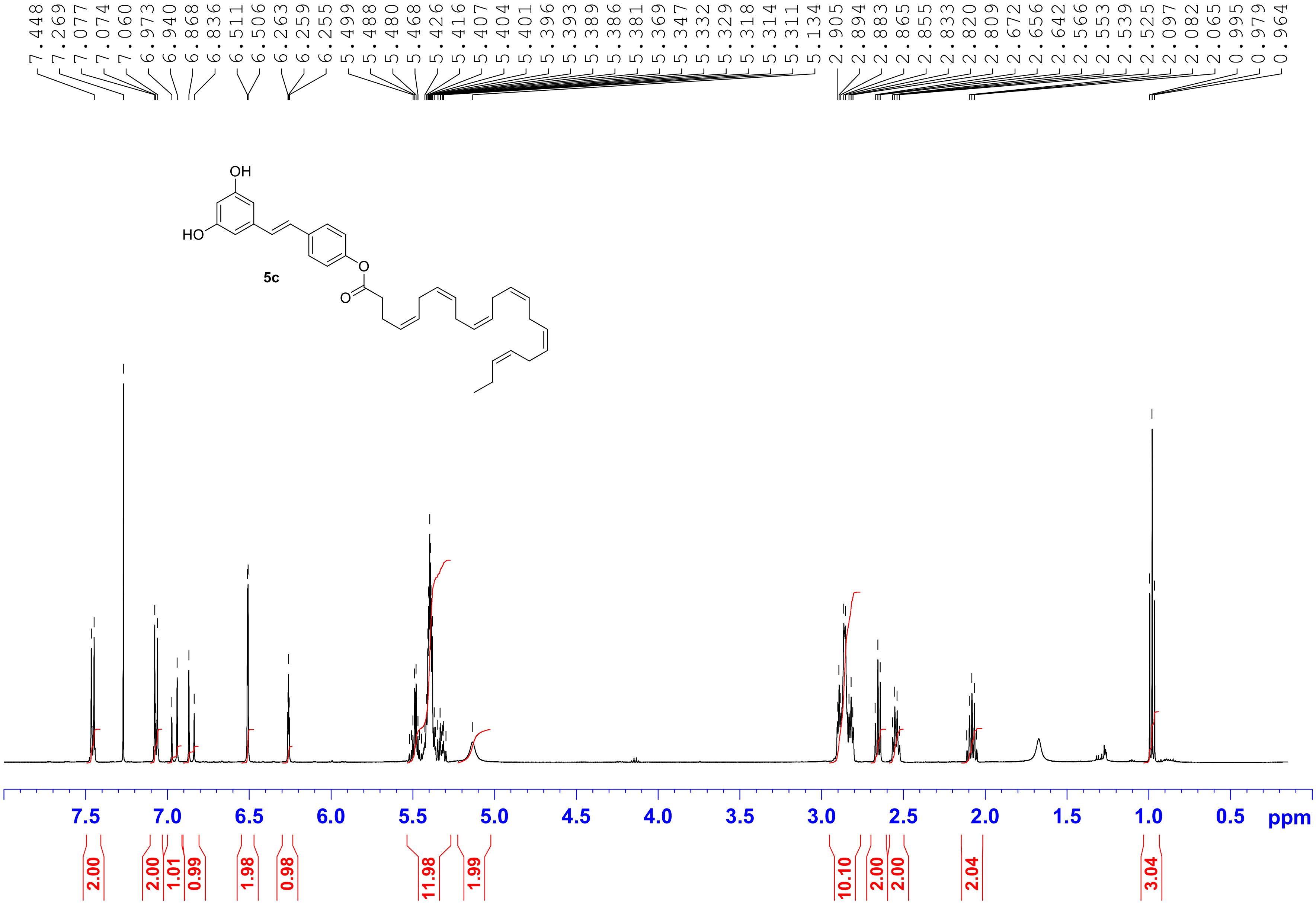
7.448
7.269
7.077
7.074
7.060
6.973
6.940
6.868
6.836
6.511
6.506
6.263
6.259
6.255
5.499
5.488
5.480
5.468
5.426
5.416
5.407
5.404
5.401
5.396
5.393
5.389
5.386
5.381
5.369
5.347
5.332
5.329
5.318
5.314
5.311
5.134
2.905
2.894
2.883
2.865
2.855
2.833
2.820
2.809
2.672
2.656
2.642
2.566
2.553
2.539
2.525
2.097
2.082
2.065
0.995
0.979
0.964
OH
HO
O
O
5c
7.5
7.0
6.5
6.0
5.5
5.0
4.5
4.0
3.5
3.0
2.5
2.0
1.5
1.0
0.5
ppm
2.00
2.00
1.01
0.99
1.98
0.98
11.98
1.99
10.10
2.00
2.00
2.04
3.04

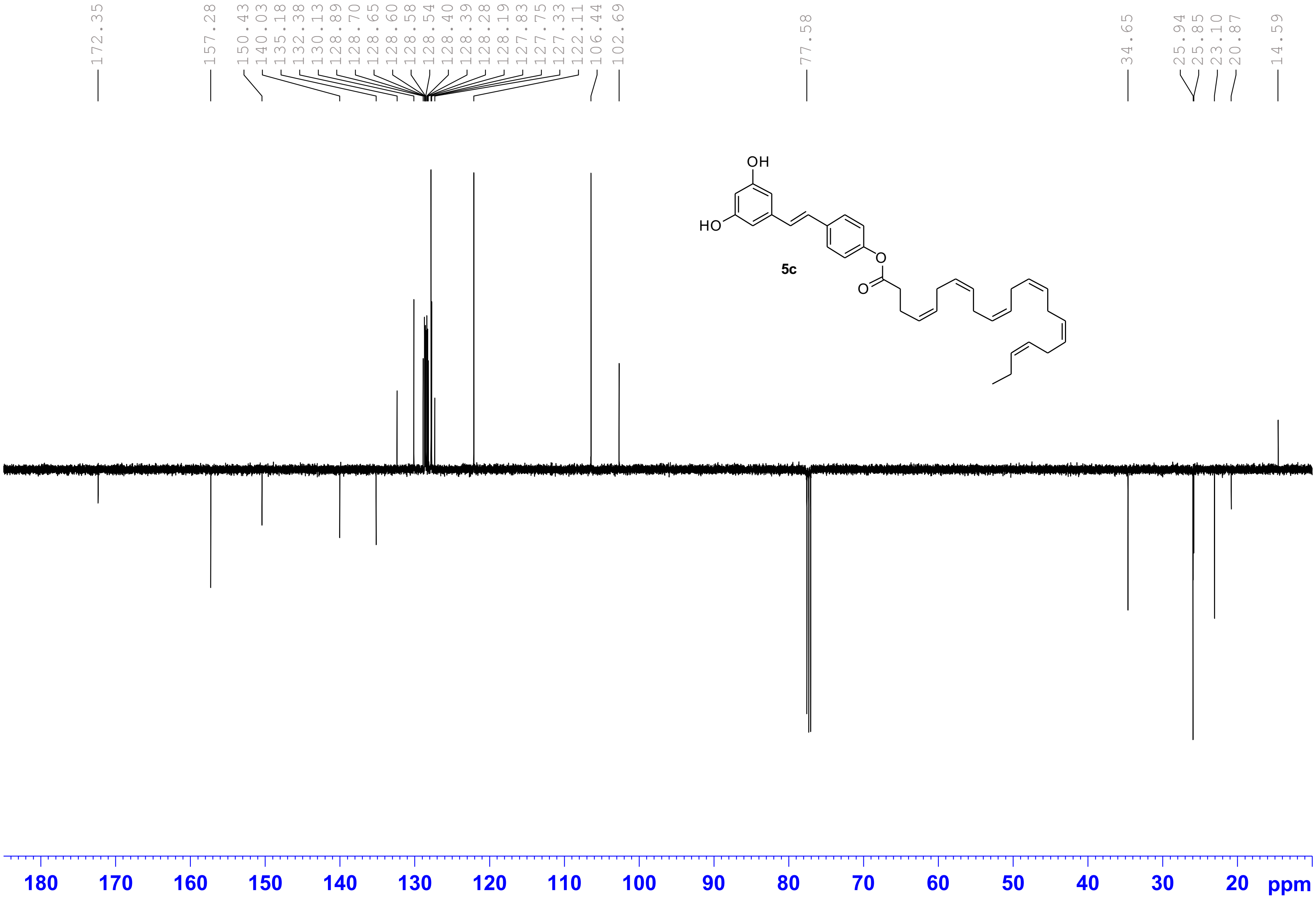

172.35
157.28
150.43
140.03
135.18
132.38
130.13
128.89
128.70
128.65
128.60
128.58
128.54
128.40
128.39
128.28
128.19
127.83
127.75
127.33
122.11
106.44
102.69
77.58
34.65
25.94
25.85
23.10
20.87
14.59
OH
HO
O
O
5c
180
170
160
150
140
130
120
110
100
90
80
70
60
50
40
30
20
ppm

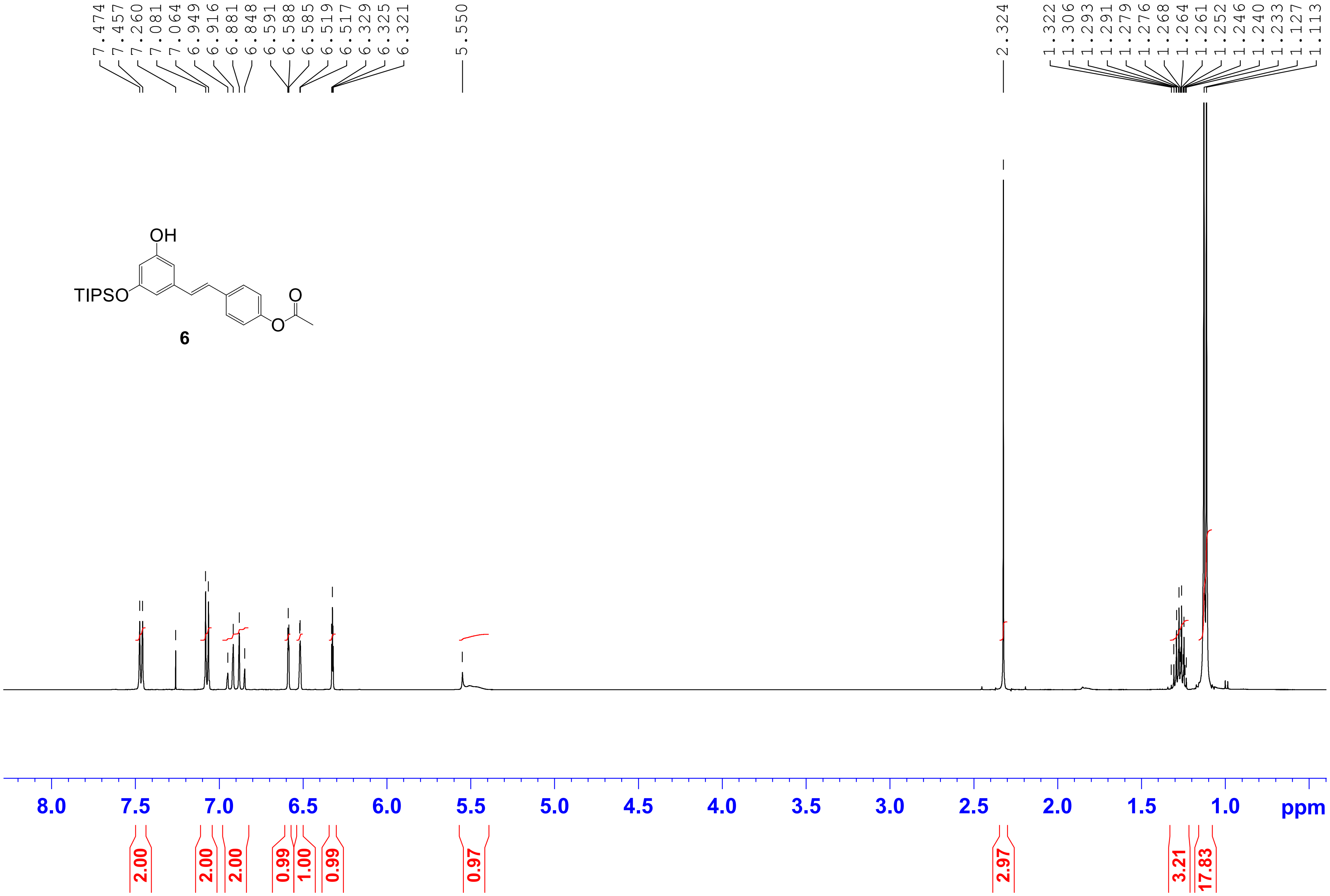
7.474
7.457
7.260
7.081
7.064
6.949
6.916
6.881
6.848
6.591
6.588
6.585
6.519
6.517
6.329
6.325
6.321
5.550
2.324
1.322
1.306
1.293
1.291
1.279
1.276
1.268
1.264
1.261
1.252
1.246
1.240
1.233
1.127
1.113
OH
TIPSO
O
O
6
8.0
7.5
7.0
6.5
6.0
5.5
5.0
4.5
4.0
3.5
3.0
2.5
2.0
1.5
1.0
ppm
2.00
2.00
2.00
0.99
1.00
0.99
0.97
2.97
3.21
17.83

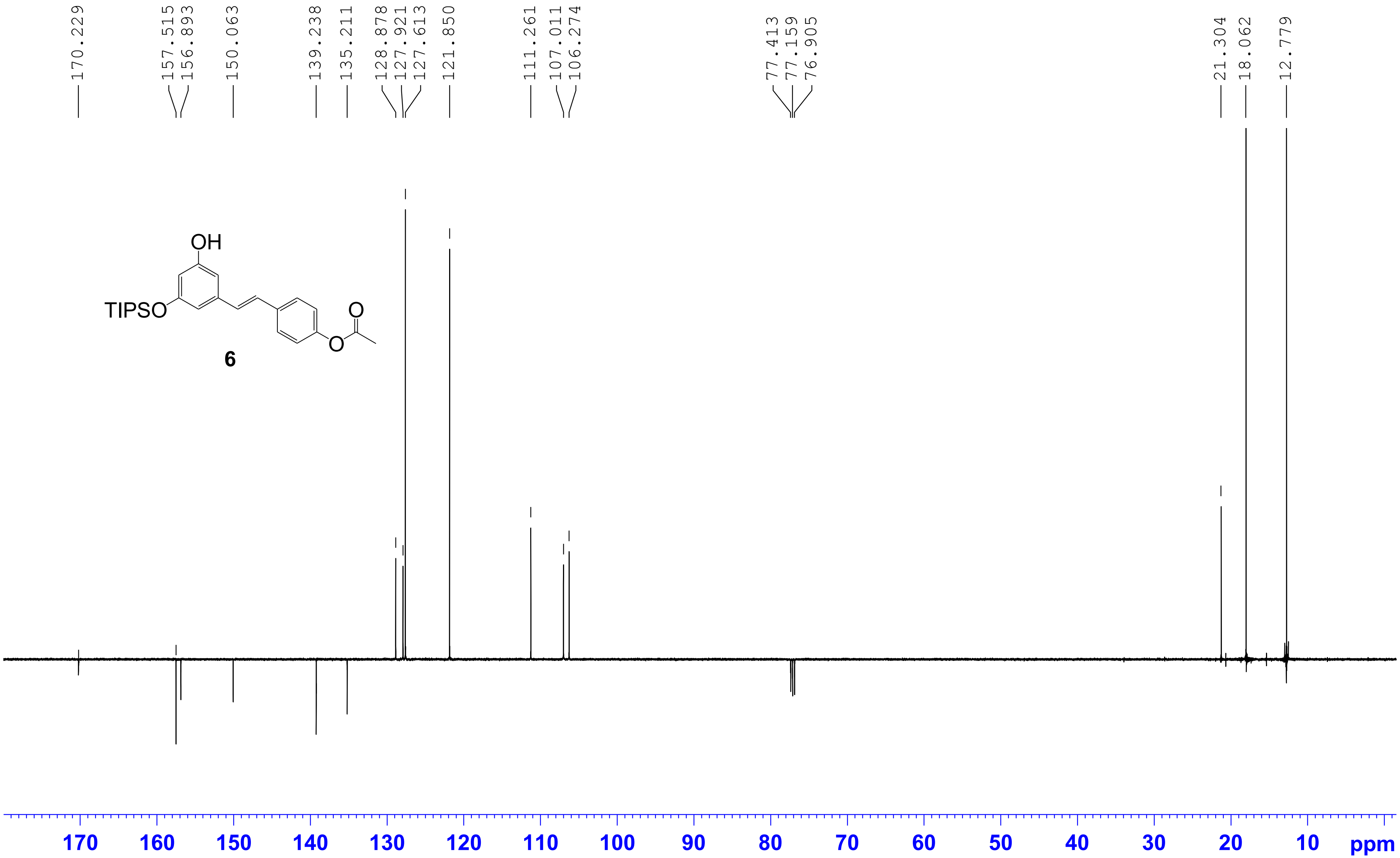
170.229
157.515
156.893
150.063
139.238
135.211
128.878
127.921
127.613
121.850
111.261
107.011
106.274
77.413
77.159
76.905
21.304
18.062
12.779
OH
TIPSO
O
O
6
170
160
150
140
130
120
110
100
90
80
70
60
50
40
30
20
10
ppm

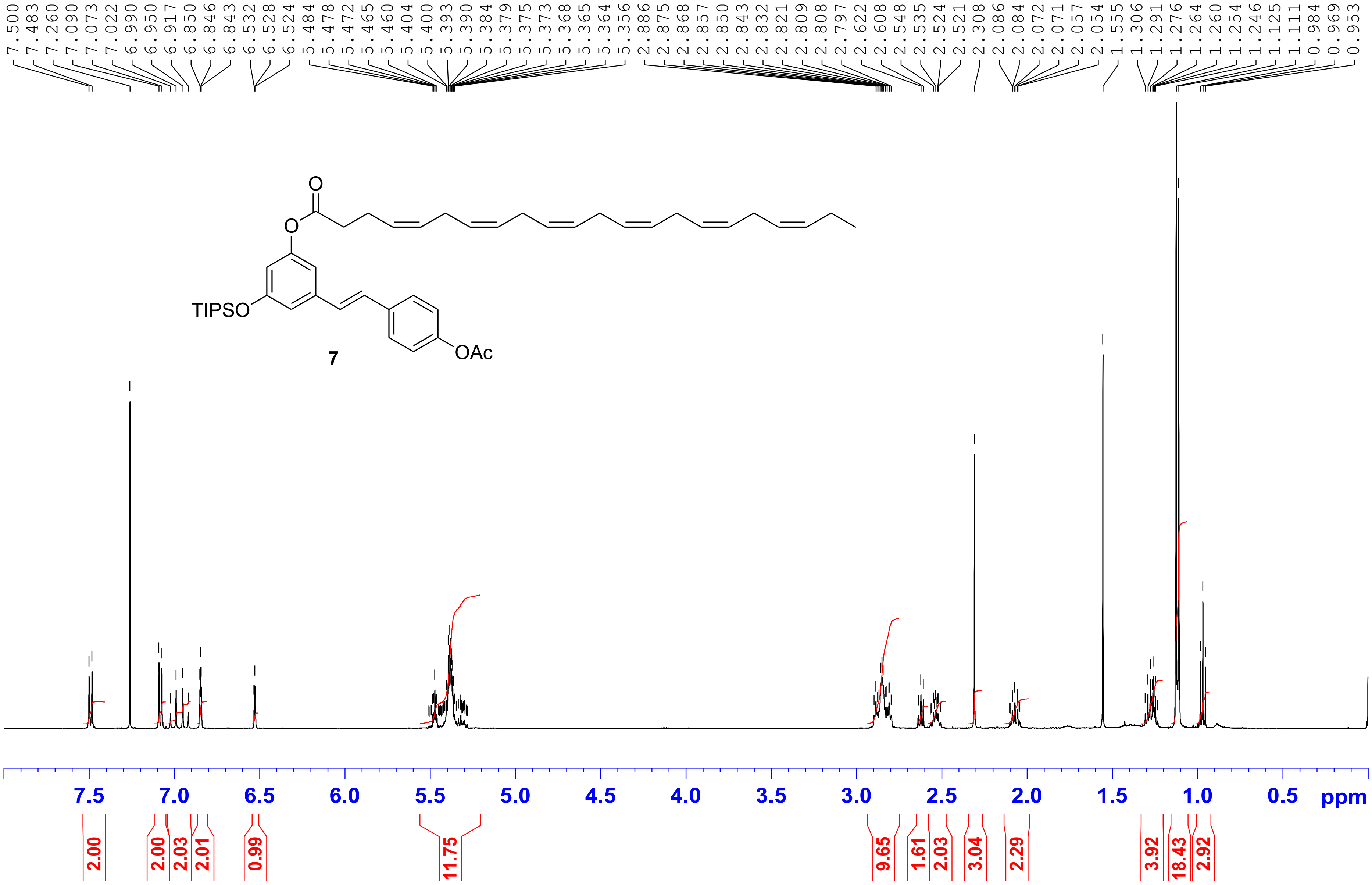

7.500
7.483
7.260
7.090
7.073
7.022
6.990
6.950
6.917
6.850
6.846
6.843
6.532
6.528
6.524
5.484
5.478
5.472
5.465
5.460
5.404
5.400
5.393
5.390
5.384
5.379
5.375
5.373
5.368
5.365
5.364
5.356
2.886
2.875
2.868
2.857
2.850
2.843
2.832
2.821
2.809
2.808
2.797
2.622
2.608
2.548
2.535
2.524
2.521
2.308
2.086
2.084
2.072
2.071
2.057
2.054
1.555
1.306
1.291
1.276
1.264
1.260
1.254
1.246
1.125
1.111
0.984
0.969
0.953
O
O
TIPSO
OAc
7
7.5
7.0
6.5
6.0
5.5
5.0
4.5
4.0
3.5
3.0
2.5
2.0
1.5
1.0
0.5
ppm
2.00
2.00
2.03
2.01
0.99
11.75
9.65
1.61
2.03
3.04
2.29
3.92
18.43
2.92

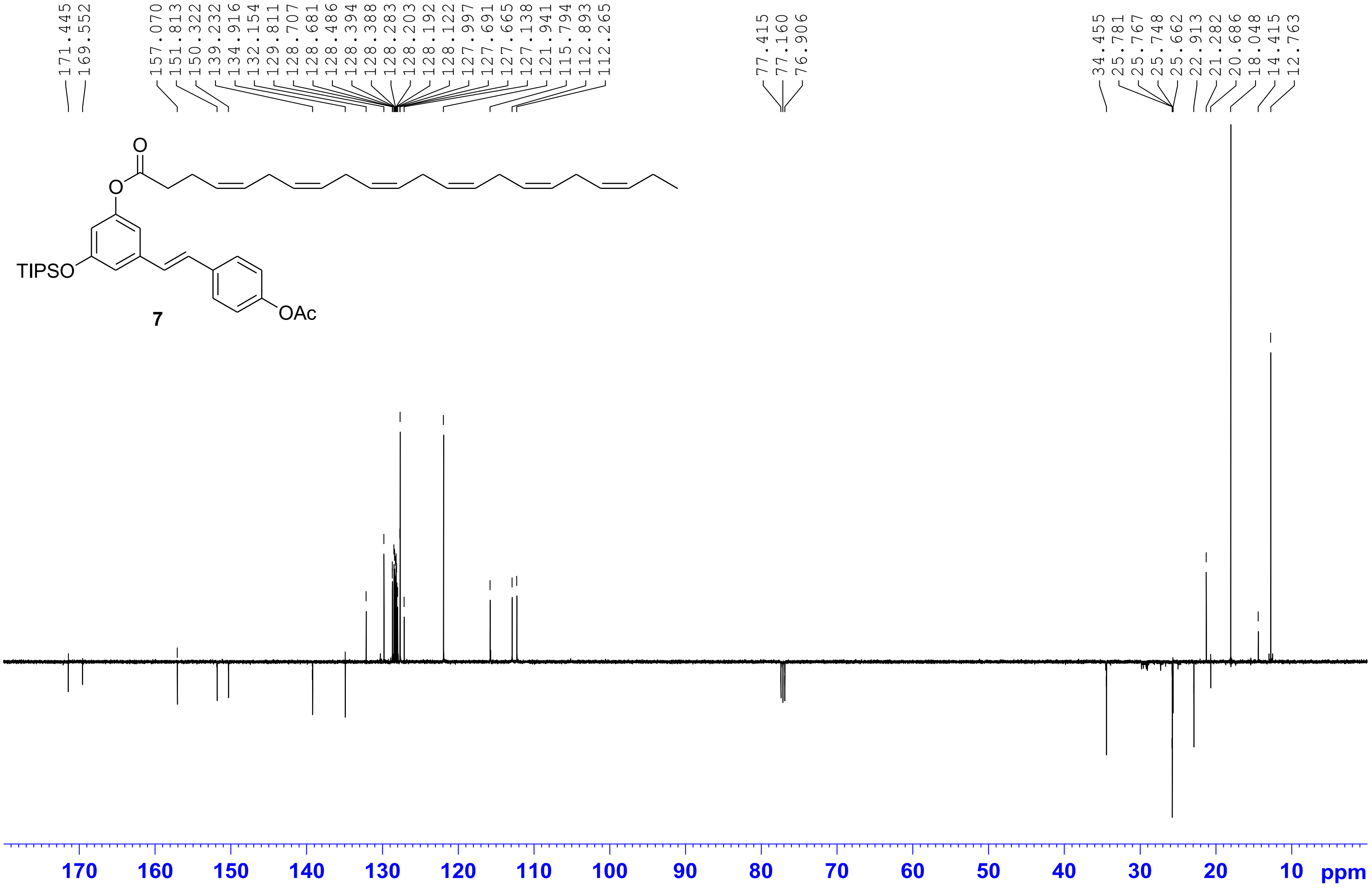
171.445
169.552
157.070
151.813
150.322
139.232
134.916
132.154
129.811
128.707
128.681
128.486
128.394
128.388
128.283
128.203
128.192
128.122
127.997
127.691
127.665
127.138
121.941
115.794
112.893
112.265
77.415
77.160
76.906
34.455
25.781
25.767
25.748
25.662
22.913
21.282
20.686
18.048
14.415
12.763
O
O
TIPSO
OAc
7
170
160
150
140
130
120
110
100
90
80
70
60
50
40
30
20
10
ppm

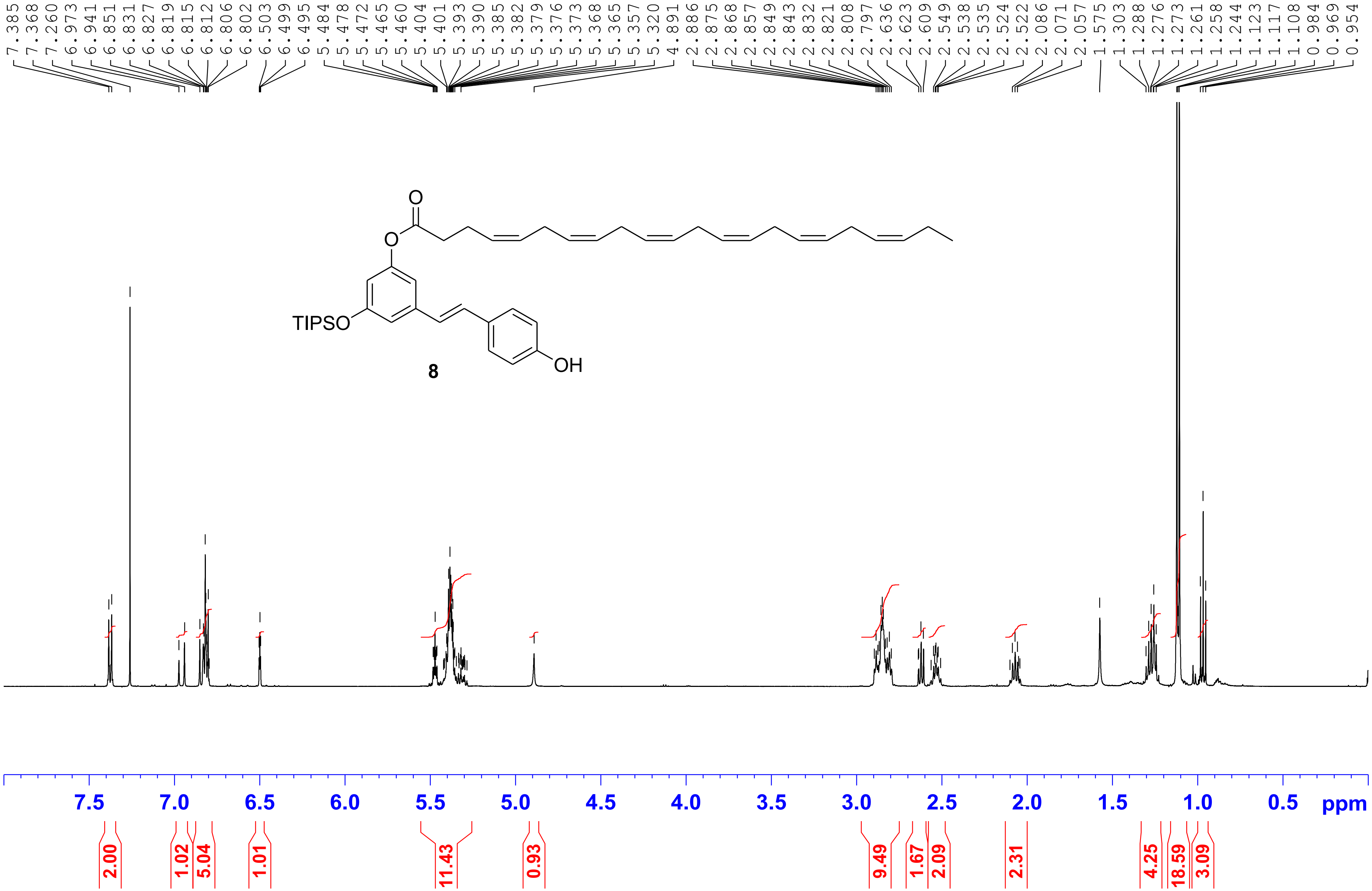
7.385
7.368
7.260
6.973
6.941
6.851
6.831
6.827
6.819
6.815
6.812
6.806
6.802
6.503
6.499
6.495
5.484
5.478
5.472
5.465
5.460
5.404
5.401
5.393
5.390
5.385
5.382
5.379
5.376
5.373
5.368
5.365
5.357
5.320
4.891
2.886
2.875
2.868
2.857
2.849
2.843
2.832
2.821
2.808
2.797
2.636
2.623
2.609
2.549
2.538
2.535
2.524
2.522
2.086
2.071
2.057
1.575
1.303
1.288
1.276
1.273
1.261
1.258
1.244
1.123
1.117
1.108
0.984
0.969
0.954
O
O
TIPSO
OH
8
7.5
7.0
6.5
6.0
5.5
5.0
4.5
4.0
3.5
3.0
2.5
2.0
1.5
1.0
0.5
ppm
2.00
1.02
5.04
1.01
11.43
0.93
9.49
1.67
2.09
2.31
4.25
18.59
3.09

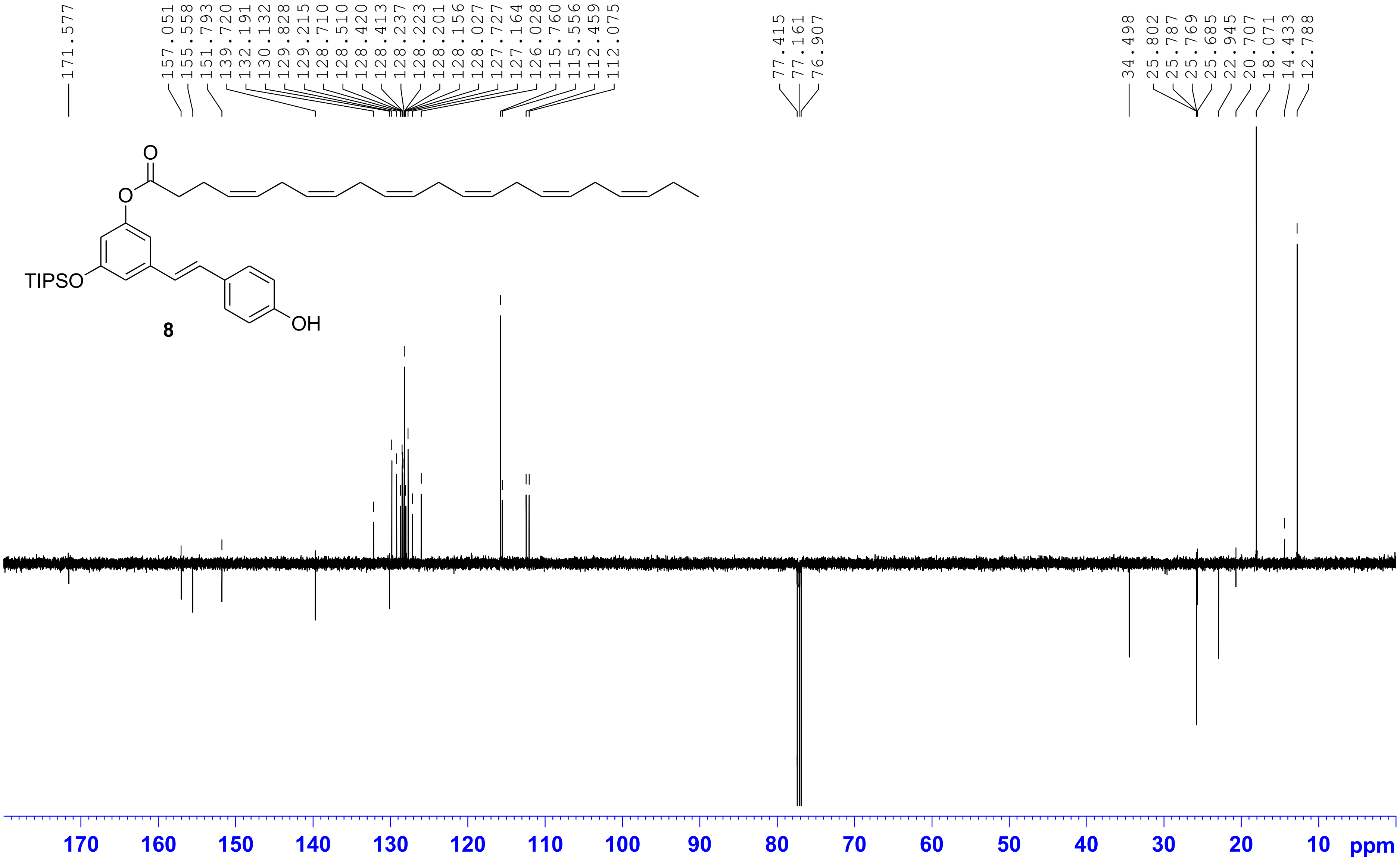

171.577
157.051
155.558
151.793
139.720
132.191
130.132
129.828
129.215
128.710
128.510
128.420
128.413
128.237
128.223
128.201
128.156
128.027
127.727
127.164
126.028
115.760
115.556
112.459
112.075
77.415
77.161
76.907
34.498
25.802
25.787
25.769
25.685
22.945
20.707
18.071
14.433
12.788
O
O
TIPSO
OH
8
170
160
150
140
130
120
110
100
90
80
70
60
50
40
30
20
10
ppm

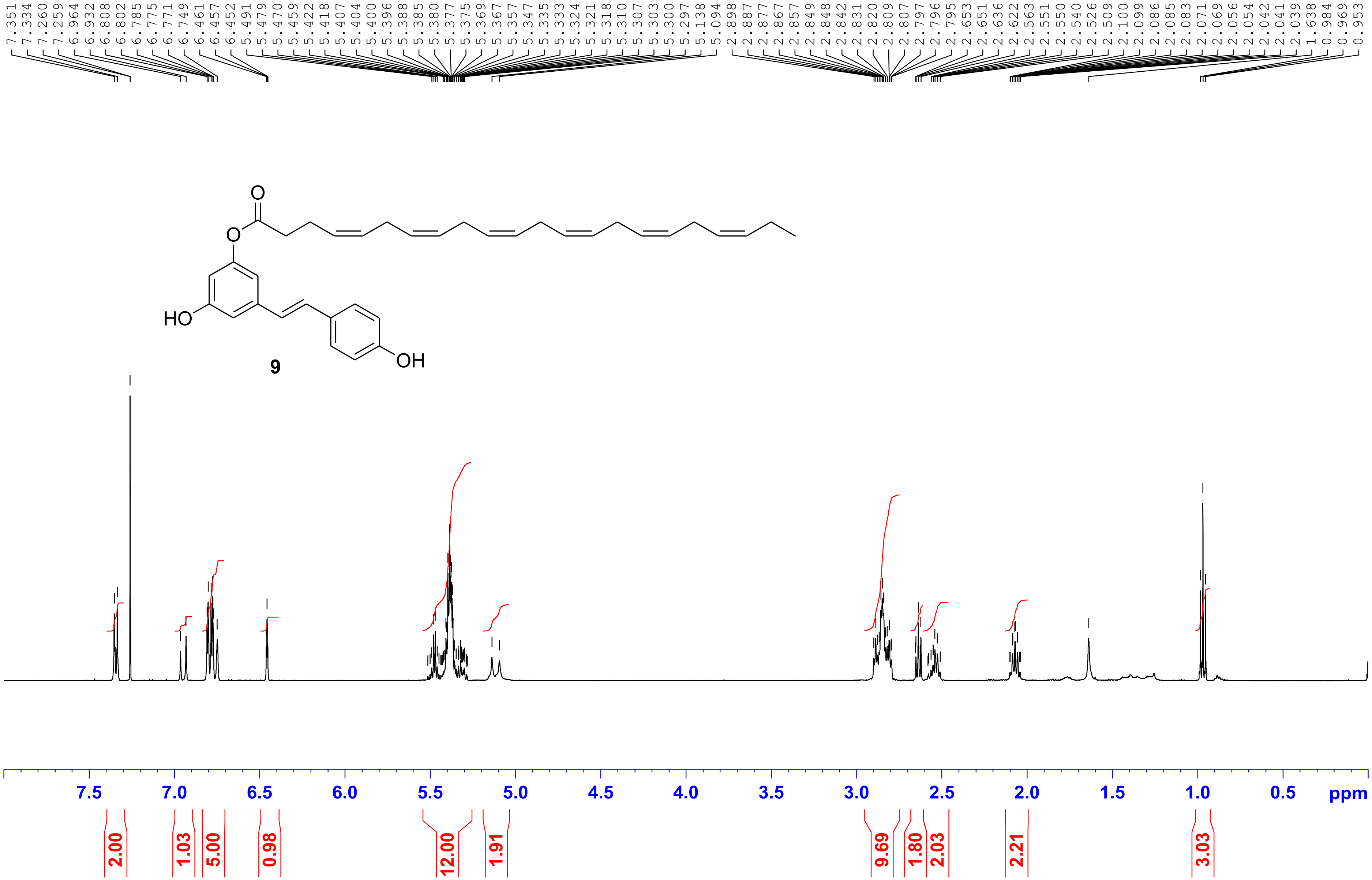

7.351
7.334
7.260
7.259
6.964
6.932
6.808
6.802
6.785
6.775
6.771
6.749
6.461
6.457
6.452
5.491
5.479
5.470
5.459
5.422
5.418
5.407
5.404
5.400
5.396
5.388
5.385
5.380
5.377
5.375
5.369
5.367
5.357
5.347
5.335
5.333
5.324
5.321
5.318
5.310
5.307
5.303
5.300
5.297
5.138
5.094
2.898
2.887
2.877
2.867
2.857
2.849
2.848
2.842
2.831
2.820
2.809
2.807
2.797
2.796
2.795
2.653
2.651
2.636
2.622
2.563
2.551
2.550
2.540
2.526
2.509
2.100
2.099
2.086
2.085
2.083
2.071
2.069
2.056
2.054
2.042
2.041
2.039
1.638
0.984
0.969
0.953
O
O
HO
OH
7.5
7.0
6.5
6.0
5.5
5.0
4.5
4.0
3.5
3.0
2.5
2.0
1.5
1.0
0.5
ppm
2.00
1.03
5.00
0.98
12.00
1.91
9.69
1.80
2.03
2.21
3.03

9

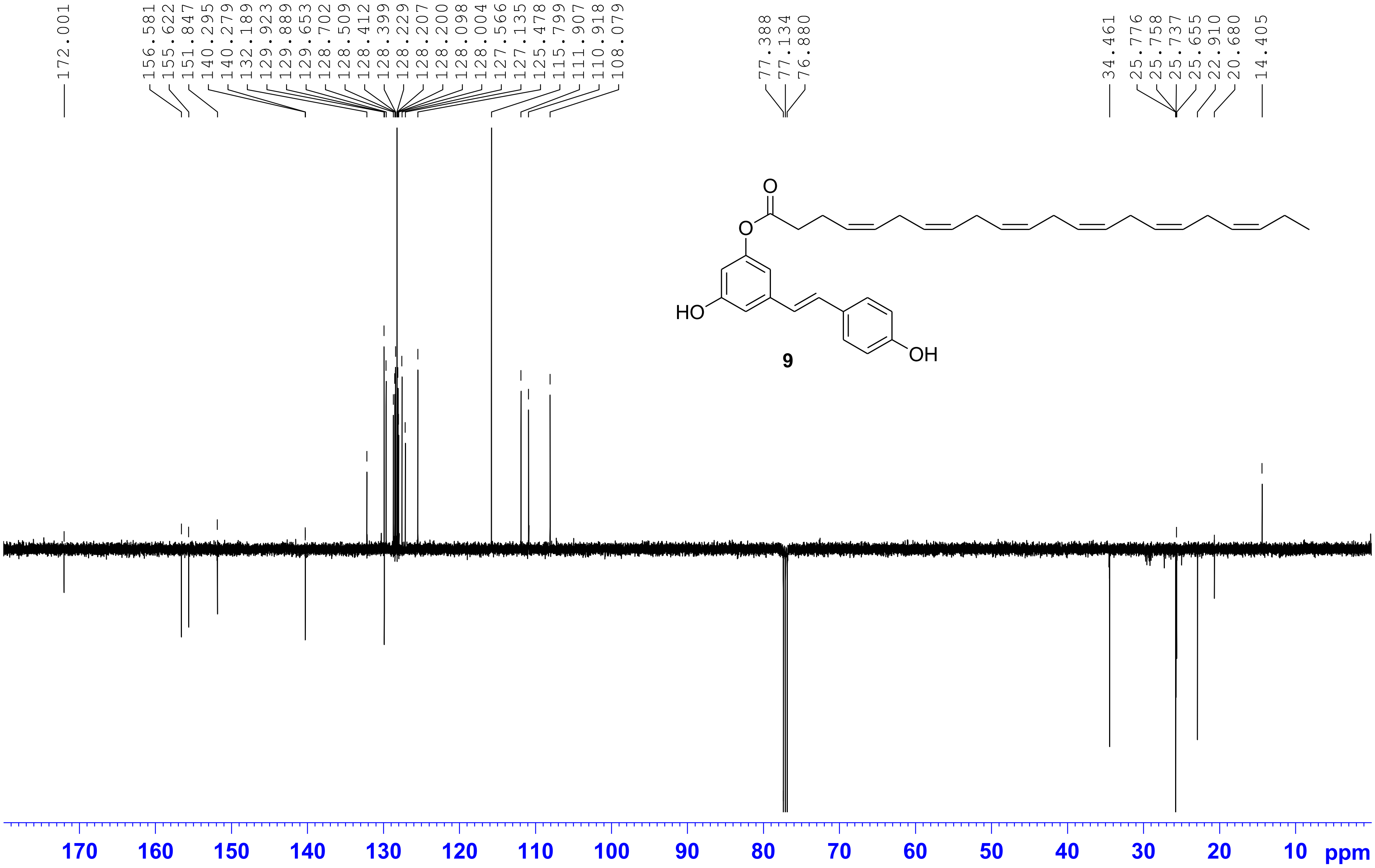

172.001
156.581
155.622
151.847
140.295
140.279
132.189
129.923
129.889
129.653
128.702
128.509
128.412
128.399
128.229
128.207
128.200
128.098
128.004
127.566
127.135
125.478
115.799
111.907
110.918
108.079
77.388
77.134
76.880
34.461
25.776
25.758
25.737
25.655
22.910
20.680
14.405
HO
OH
O
O
9
170 160 150 140 130 120 110 100 90 80 70 60 50 40 30 20 10 ppm

Phosphokinase status of THP-1 (untreated group)

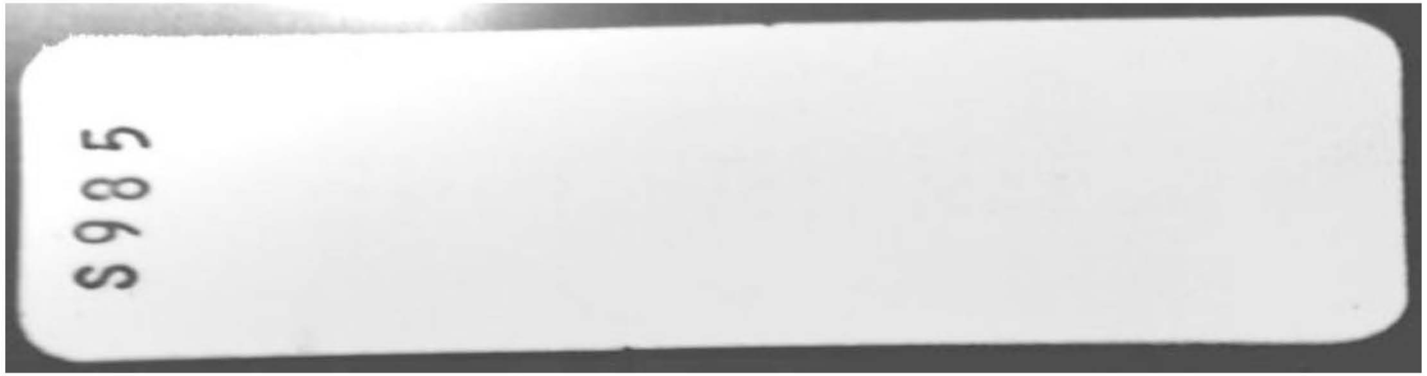

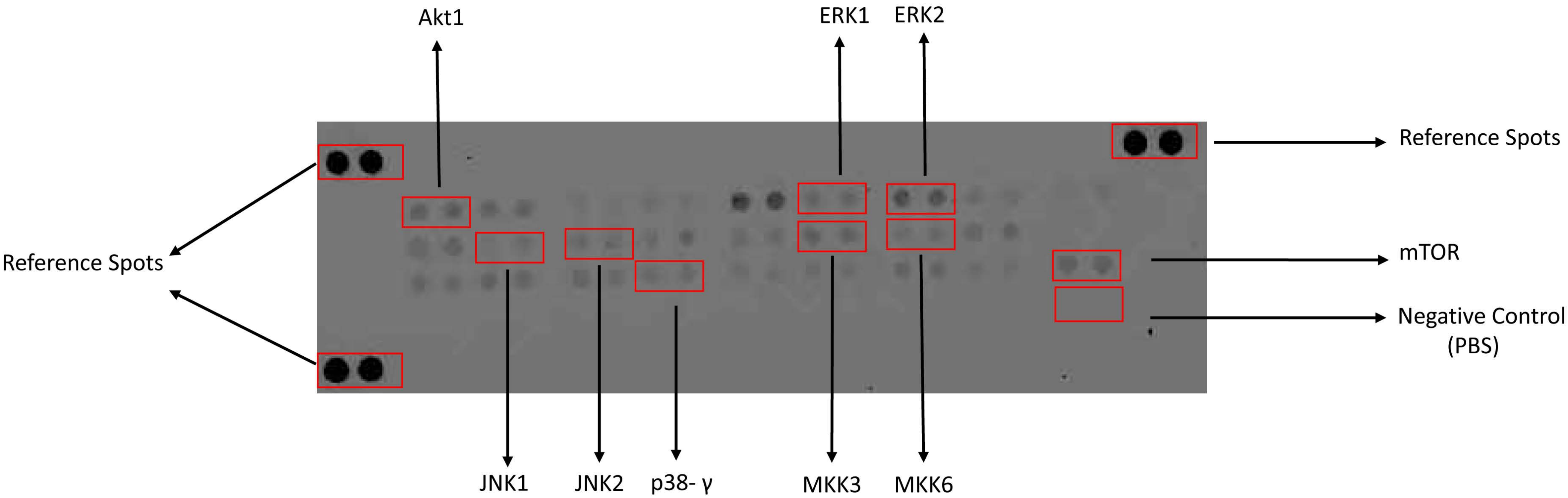

Phosphokinase status of THP-1
(TNF-α treated group)

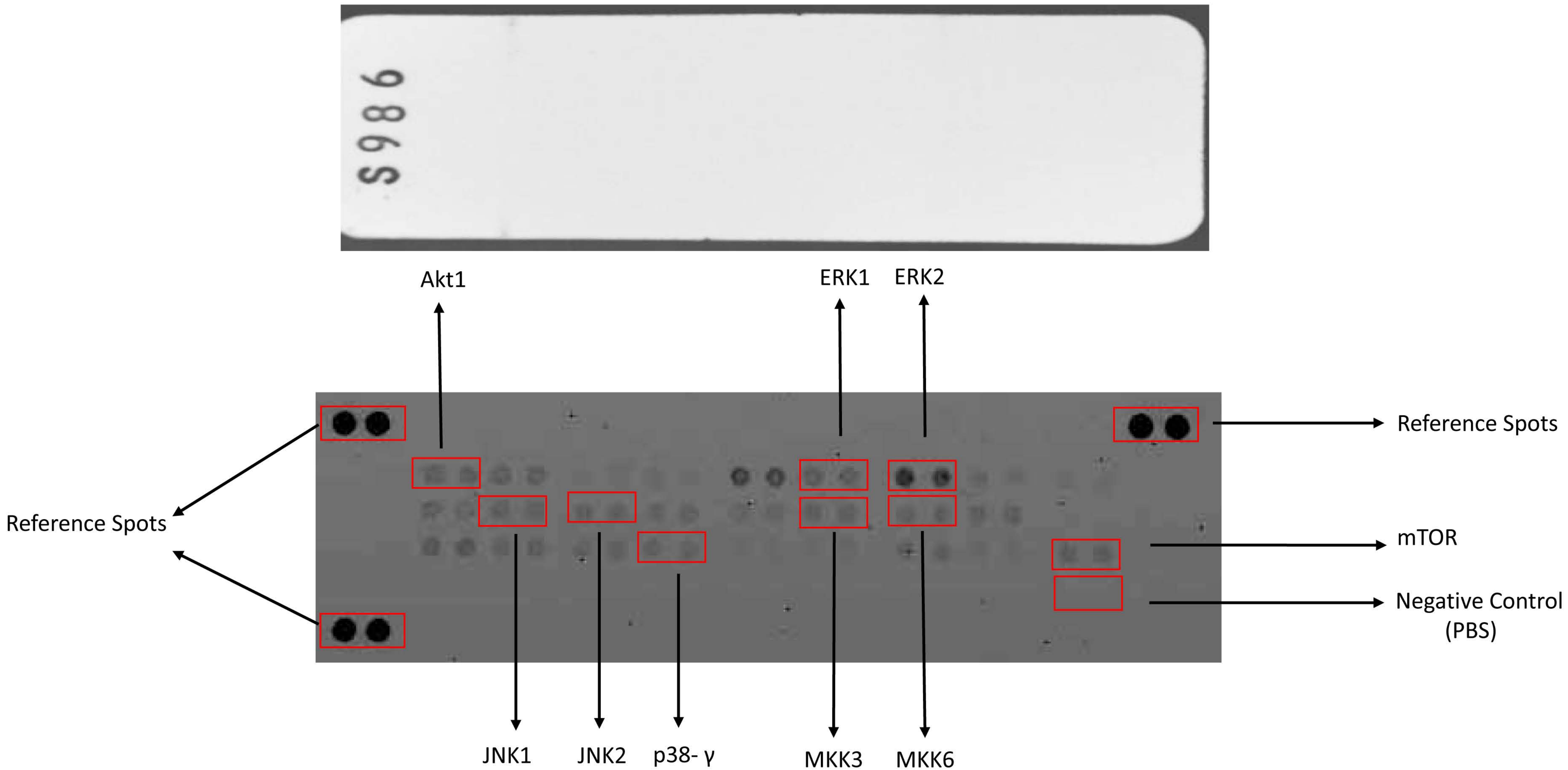

Phosphokinase status of THP-1 (TNF-α + RES)

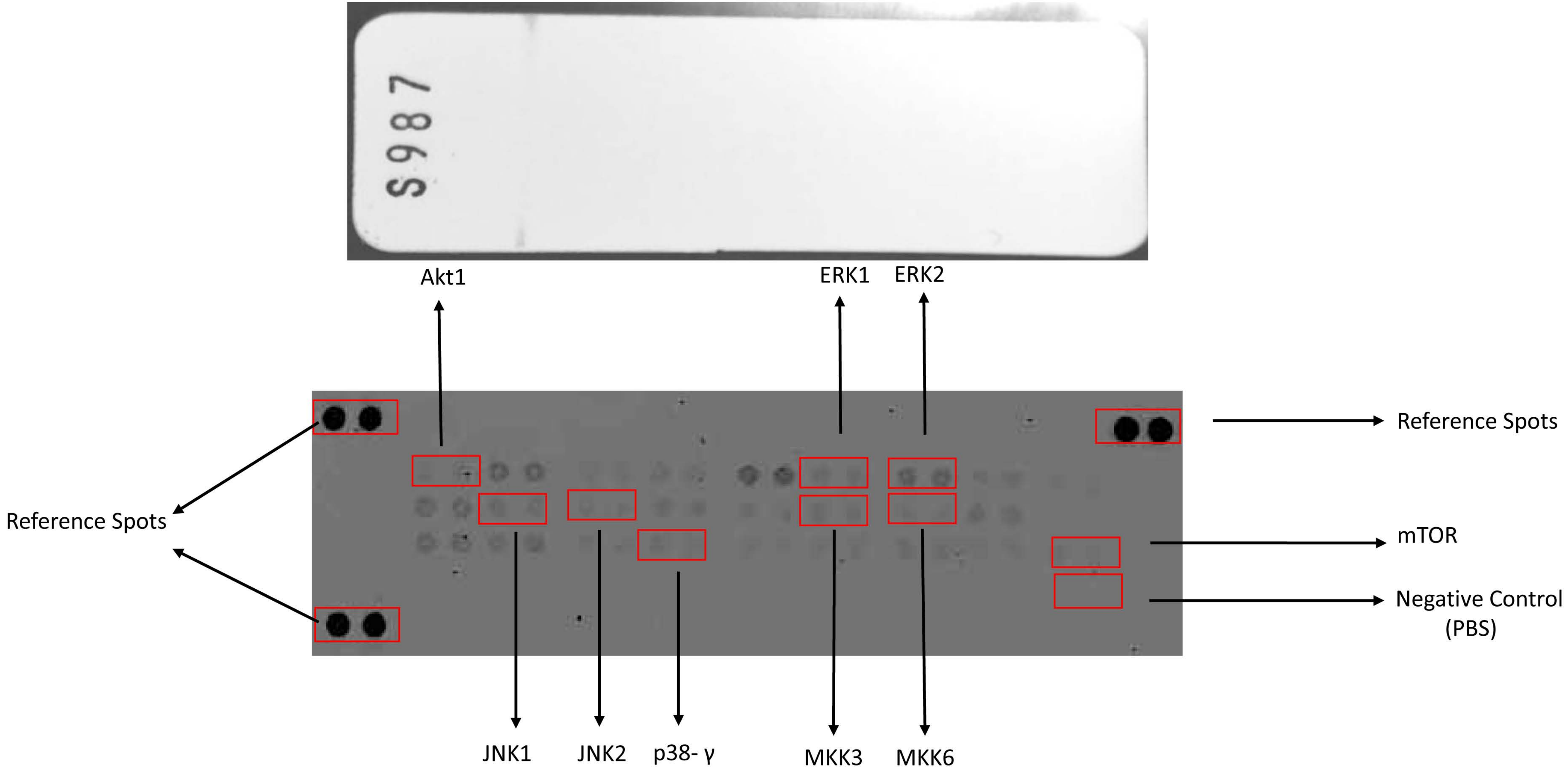

Phosphokinase status of THP-1 (TNF-α + RES-LA)

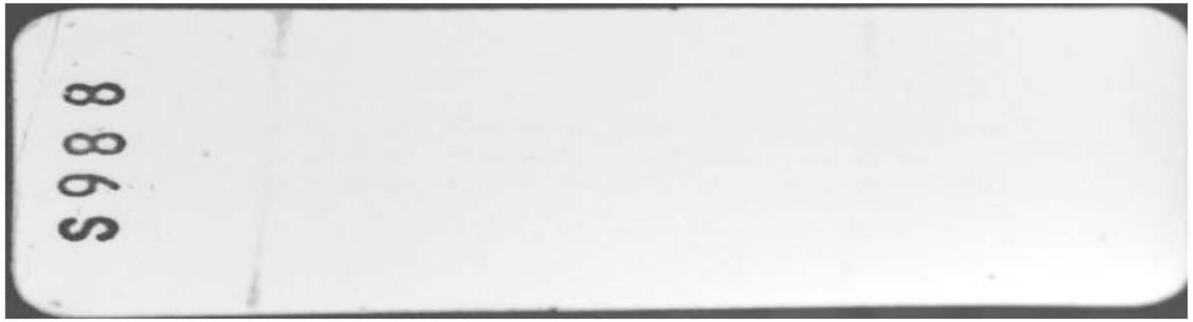

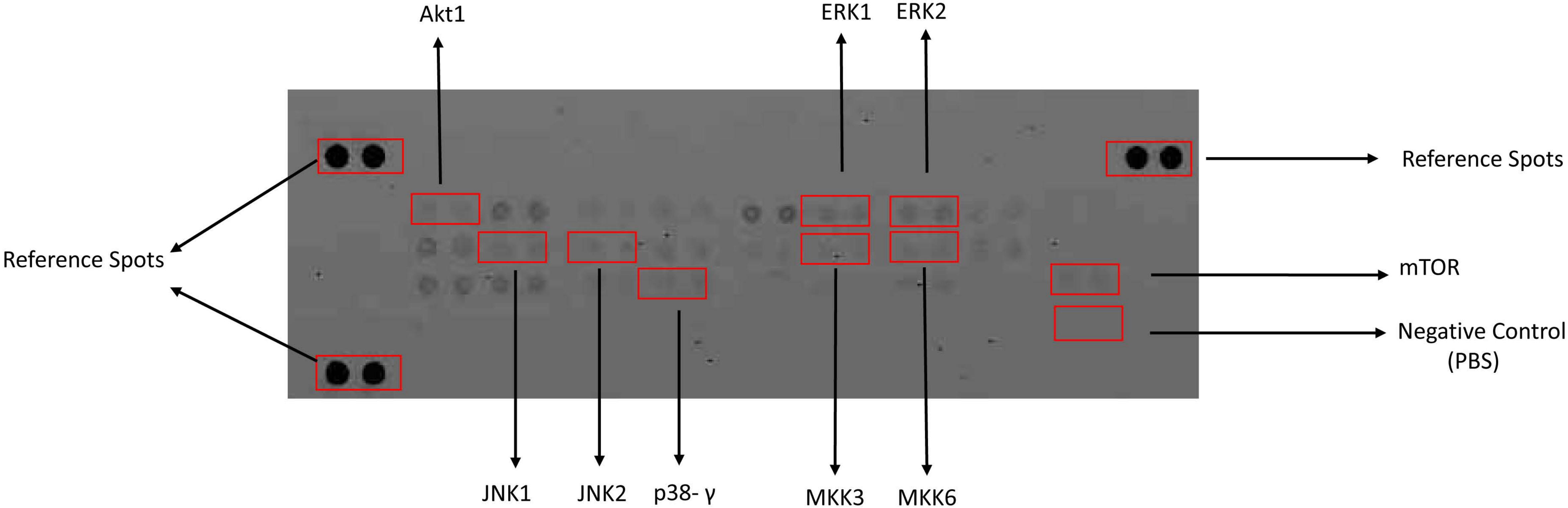

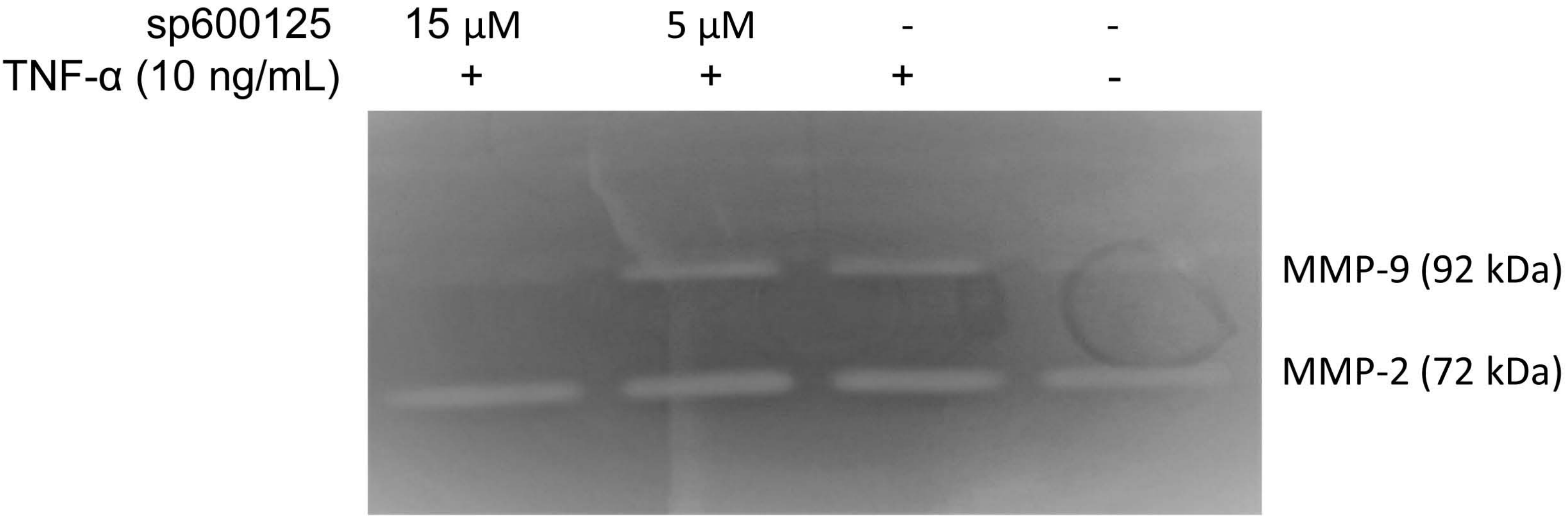
sp600125 15 μM 5 μM - -
TNF-α (10 ng/mL) + + + -
MMP-9 (92 kDa)
MMP-2 (72 kDa)
